# Seasonal Variations in the Microbiome of *Hyalomma excavatum* Ticks in Algeria

**DOI:** 10.1007/s00248-025-02597-y

**Published:** 2025-09-30

**Authors:** Salma Kaoutar Abdelali, Lynda Aissaoui, Ana Laura Cano-Argüelles, Elianne Piloto-Sardiñas, Lianet Abuin-Denis, Apolline Maitre, Angélique Foucault-Simonin, Lourdes Mateos-Hernández, Myriam Kratou, Alejandra Wu-Chuang, Dasiel Obregon, Alejandro Cabezas-Cruz

**Affiliations:** 1 Laboratory of Research on the Improvement and Development of Animal and Plant Production, University of Ferhat Abbas, Setif, Algeria; 2 Parasitology Laboratory, Institute of Natural Resources and Agrobiology of Salamanca, IRNASA, CSIC, Cordel de Merinas, 40-52, 37008 Salamanca, Spain; 3 UMR BIPAR, Laboratoire de Santé Animale, ANSES, INRAE, Ecole Nationale Vétérinaire d’Alfort, 94700 Maisons-Alfort, France; 4 Direction of Animal Health, National Center for Animal and Plant Health, Carretera de Tapaste y Autopista Nacional, Apartado Postal 10, 32700 San José de Las Lajas, Mayabeque Cuba; 5 Animal Biotechnology Department, Center for Genetic Engineering and Biotechnology, Avenue 31 Between 158 and 190, P.O. Box 6162, Havana, 10600 Cuba; 6 Laboratory of Microbiology, National School of Veterinary Medicine of Sidi Thabet, University of Manouba, Manouba, 2010 Tunisia; 7 School of Environmental Sciences, University of Guelph, Guelph, ON N1G 2W1 Canada

**Keywords:** *Hyalomma excavatum*, Bacterial diversity, Microbiome interactions, Seasonal variation, Next-generation sequencing (NGS), Vector ecology

## Abstract

Ticks are key vectors of zoonotic diseases, with their microbiomes playing a critical role in tick physiology, survival, and vector competence. This study presents the first investigation of the microbiome in *Hyalomma excavatum* ticks from Algeria, focusing on seasonal variations in bacterial diversity, community composition, and pathogen interactions. Using next-generation sequencing (NGS), the microbiome of 21 female ticks collected during spring, summer, and autumn was analyzed. Beta diversity analysis revealed significant seasonal shifts in microbial community structure, while alpha diversity metrics showed no significant differences in richness and evenness. Co-occurrence network analysis demonstrated seasonal shifts in microbial interactions, particularly between symbionts and pathogens, highlighting *Francisella* as a key taxon in tick survival and pathogen dynamics. *Rickettsia* presence varied by season, influencing microbial network stability. These findings underscore the ecological determinants shaping the microbiome and its potential role in pathogen transmission. Understanding seasonal microbiome shifts provides valuable insights for managing tick-borne diseases and could inform the development of targeted, season-specific vector control strategies.

**Supplementary Information:**

The online version contains supplementary material available at 10.1007/s00248-025-02597-y.

## Introduction

Ticks are major vectors of zoonotic diseases, transmitting a wide range of pathogens that pose significant threats to both human and animal health [3, 39]. Of the nearly 900 known tick species, the majority fall into two main families: Ixodidae (hard ticks) and Argasidae (soft ticks) [18, 23]. As obligate blood-feeding arthropods [9], their ability to acquire, harbor, and transmit pathogens is influenced by biological, environmental, and microbial factors [5, 13].

In Algeria, species from the genus *Hyalomma* are prevalent, particularly *Hyalomma excavatum*, which dominates arid and semi-arid regions [12, 17, 43, 47]. These ticks serve as vectors for zoonotic and livestock pathogens, including *Theileria annulata*,* Babesia bovis*, *B. bigemina*, and *B. divergens* (causative agents of babesiosis); *Anaplasma marginale*, *A. phagocytophilum*, *A. centrale*, and *A. bovis*; *Ehrlichia* spp.; *Candidatus* Neoehrlichia; as well as several *Rickettsia* species such as *R. aeschlimannii*, *R. massiliae*, *R. monacensis*, *R. africae*, *R. sibirica* subsp. *mongolitimonae*, *R. slovaca*, *R. helvetica*, and *R. felis*, which are of public health concern [6, 16, 22, 37, 48] and contribute to economic losses in Algeria’s livestock industry.

Ticks harbor one of the most diverse microbiomes among arthropods [7], which includes not only a wide array of symbiotic, opportunistic, and environmentally acquired bacteria, but also diverse communities of viruses, fungi, and protozoa components [44]. Numerous studies have shown that these microbial communities are not passive inhabitants but actively contribute to vector competence [29, 45], tick physiology [24], and survival [11]. For instance, *Francisella-*like endosymbionts (FLEs) contribute to tick survival by supplementing nutrients, particularly B vitamins (biotin, riboflavin, and folate) and amino acids like cysteine, threonine, tyrosine, tryptophan, phenylalanine, and serine from pyruvate, which are deficient in blood meals [24, 42]. Additionally, FLEs influence microbial community structure, underlining their potential as targets for innovative strategies for tick and pathogen control [40]. Despite the importance of *Hyalomma* ticks, their microbiomes remain understudied, particularly in the unique ecological contexts of North Africa. Therefore, it is crucial to examine the role of pathogens like *Rickettsia* and symbionts such as FLEs within these microbial communities. For instance, in *Rhipicephalus microplus*, *A. marginale* interacts with other microbiome members, influencing disease transmission and vector competence [33]. Rickettsial pathogens also shape microbiota assembly in *Hyalomma marginatum*, *Rhipicephalus bursa*, *R. sanguineus*, and *R. turanicus* ticks [27, 28] illustrating the complexity of pathogen–microbiome relationships*.*

Investigating the effect of seasonal dynamics on microbial diversity in ticks remains crucial [34]. Abiotic factors, such as temperature, influence the microbial community structure associated with *Hyalomma dromedarii* [31], emphasizing the importance of investigating seasonal microbiome shifts to enhance tick control strategies. Recent studies suggest that climate change can shape the ecological dynamics of FLEs in tick microbiomes, with potential implications for vector competence [40]. Together, these insights underscore the need to investigate microbiome dynamics in *Hyalomma* ticks, not only to address knowledge gaps but also to inform strategies for tick and pathogen control in Algeria’s underrepresented ecosystems.

Given the potential influence of seasonal and ecological factors on tick-associated microbiota, this study explores how seasonal variations shape the microbial communities of *H. excavatum*, with a particular focus on the interactions between *Rickettsia*, FLE, and other microbial taxa. We hypothesized that seasonal shifts modulate microbial interactions within the tick microbiome, potentially altering the ecological roles and co-occurrence patterns of key taxa. To test this, the microbiomes of *H. excavatum* ticks positive for *Rickettsia* and *Francisella* were examined using next-generation sequencing, and interaction networks were analyzed to uncover seasonal patterns and the structural importance of individual taxa.

## Material and Methods

### Study Design and Tick Samples

This study investigated the bacterial diversity within the microbiome of *Hyalomma excavatum* ticks collected from untreated cattle across three seasons: spring (March to May), summer (June to August), and autumn (September to November). A total of 21 engorged female tick samples (autumn: 8, spring: 7, summer: 6) were analyzed, corresponding to those previously described by Abdelali et al. [1]. These ticks were collected throughout 2021–2022 from local untreated breed cattle in Djelfa, Algeria (34°40′00″N, 3°15′00″E), a region recognized as the steppe capital of the country. Tick collection involved only non-invasive removal from owned animals and did not require formal ethical clearance, but was conducted with the full consent of livestock owners and in line with institutional standards for animal welfare.

### 16S rRNA Amplicon Sequencing and Processing of Raw Sequences

DNA was extracted using the NucleoSpin Tissue Kit (Macherey–Nagel, Germany), following the manufacturer’s protocol. The extracted DNA was quantified using a NanoDrop spectrophotometer and assessed for quality by electrophoresis on a 1.5% agarose gel, with visualization under UV light. High molecular weight DNA bands (~ 10–20 kb**)** were expected, confirming the integrity of the genomic DNA. The bacterial community was characterized by amplifying the V3–V4 region of the 16S rRNA gene using Illumina-tailed universal primers 341 F (5′ TCGTCGGCAGCGTCAGATGTGTATAA GAGACAGCCT ACGGGNGGCWGCAG-3′) and 805R (5′-GTCTCGTGGGCTCGGAGATGTGTATAA GAGACAGGACTACHVGGGTATCTAATCC-3′). Amplicons were sequenced on an Illumina MiSeq platform (Illumina, USA) with paired-end reads (2 × 250 bp) at Eurofins Genomics. All raw sequencing reads have been deposited in the NCBI Sequence Read Archive (SRA) under project number [PRJNA1214082]. Raw sequences were processed using Quantitative Insights Into Microbial Ecology, version 2023.2 (QIIME2). Quality filtering, chimera removal, and feature clustering were performed to obtain amplicon sequence variants (ASVs). Taxonomic assignments were conducted using the SILVA database (v138) [35]. Taxa contaminants were filtered using Decontam [14] in R 4.3.3 [36] via RStudio 2023.06.1 [38], applying a 0.5 probability threshold and the “prevalence” method to remove likely false reads. This approach compares the prevalence of each sequence feature in true samples with its prevalence in negative controls, allowing for the identification and removal of contaminants. In addition, taxa with fewer than three reads in any sample were excluded to focus on relevant microorganisms. Alpha-diversity and beta-diversity metrics were calculated to assess microbial richness and composition. Co-occurrence network analysis was applied to explore interactions between bacterial taxa, with a particular focus on the roles of *Rickettsia* and *Francisella*.

### Microbial Diversity, Composition, and Taxonomic Differential Relative Abundance

To investigate changes in the microbiome across three seasons, both alpha and beta diversity metrics were analyzed using QIIME 2 software [8]. Alpha diversity was evaluated to measure community richness and evenness, using the metrics observed features [15], Faith’s phylogenetic diversity index [19], Pielou’s evenness index [32], and Shannon’s diversity index (Shannon, 1948). Group comparisons for alpha diversity metrics were performed using the Kruskal–Wallis test (*p* ≤ 0.05). Beta diversity was measured using the Bray–Curtis dissimilarity index [10], and to assess differences between specific groups pairwise PERMANOVA test (*p* ≤ 0.05) was conducted. Additionally, beta dispersion was analyzed with the “betadisper” function in the Vegan package [30] in R version 4.3.3 [36] implemented in RStudio version 2023.06.1[38], while the statistical significance was tested with ANOVA (*p* ≤ 0.05). In addition, cluster analyses were carried out using the Jaccard similarity coefficient in Vegan. Unique versus shared taxa among the three conditions were visualized using Venn diagrams generated with the Venn Diagram web tool (https://bioinformatics.psb.ugent.be/webtools/Venn/).

Differential taxonomic abundance was assessed using the ALDEx2 method [20] in R version 4.3.3 [36] conducted in RStudio version 2023.06.1 [38] and significant differences between groups were identified with the Kruskal–Wallis test (*p* ≤ 0.05). Heatmaps illustrating differentially abundant taxa (with relative abundance transformed using the centered log-ratio (clr)) were created using the “Heatplus” package in R version 4.3.3 [36] performed in RStudio version 2023.06.1 [38]. These analyses provided insights into the dynamic composition of the microbiome and highlighted taxa that varied significantly across the tested conditions.

### Inference of Bacterial Co-occurrence Networks

To explore interactions within microbial communities, co-occurrence networks were built using taxonomic profiles at the genus level. The Sparse Correlations for Compositional Data (SparCC) method [21] was employed in R version 4.3.3 [36], performed in RStudio version 2023.06.1 [38]. Empirical *p*- values were calculated using a bootstrap approach with 1,000 iterations and adjusted for multiple testing using the False Discovery Rate (FDR) method. Only statistically significant correlations (adjusted *p* < 0.05) with an absolute value ≥ 0.75 were retained. A conservative threshold of |*r*|≥ 0.75 was chosen to minimize spurious associations and highlight the most reliable interactions. In the resulting networks, nodes represent individual taxa and edges indicate significant positive (*r* ≥ 0.75) or negative (*r* ≤ – 0.75) associations.

Key topological metrics were computed and visualized using Gephi version 0.9.5 [4]. These metrics included the total number of nodes and edges, network diameter (measuring the shortest path between the most distant nodes), modularity (quantifying the degree of division into modules), average degree (mean number of edges per node), weighted degree (sum of edge weights per node), and clustering coefficient (indicating the likelihood of nodes forming tightly connected groups) [2].

### Subnetwork Analysis of *Rickettsia* and *Francisella* Associations

To explore the interactions of *Rickettsia* and *Francisella* within the microbial community, their direct associations with other bacterial taxa were analyzed. Sub-networks were constructed to highlight these positive and negative connections, providing a focused visualization of their immediate relationships. The analyses were performed using Gephi version 0.9.5 [4], with edge strengths represented by SparCC correlation weights. Sub-networks were extracted using the ego-network filtering function in Gephi version 0.9.5 [4], selecting first-order neighbors of each focal taxon.

### Microbial Network Robustness Analysis

To evaluate the robustness of microbial co-occurrence networks under perturbations, the impact of node removal or addition on network connectivity was examined. The proportion of nodes that needed to be removed to reduce connectivity by 80% was determined. This analysis included random and directed node removal based on betweenness centrality (removing nodes with the highest betweenness centrality), degree centrality (removing nodes with the highest degree), and cascading removal (recalculating betweenness centrality after each node removal). The Network Strengths and Weaknesses Analysis (NetSwan) package [26] in R version 4.3.3 [36] was used for this assessment, conducted within the RStudio version 2023.06.1 [38].

Additionally, a node addition analysis was performed to simulate ecologically relevant scenarios, such as the introduction of new microbial taxa over time or across different environmental conditions. In each simulation, 100, 300, 500, 700, or 1000 nodes were randomly added to the existing network, and their integration patterns were analyzed. The size of the largest connected component (LCC)—i.e., the number of nodes in the biggest cluster—and the average path length (APL)—the average number of steps along the shortest paths for all possible node pairs—were calculated to assess the effects of increasing microbial richness on network structure and resilience. The results were visualized using GraphPad Prism 9 software (GraphPad Software Inc., San Diego, CA, USA).

### Statistical Analysis

Alpha diversity differences between groups were evaluated using the Kruskal–Wallis test (*p* < 0.05) in QIIME2 version 2023.2 [8]. The Bray–Curtis dissimilarity index was used to compare group differences, with statistical significance determined by a pairwise PERMANOVA test (*p* < 0.05). Beta dispersion was assessed with an ANOVA test (*p* < 0.05). Taxa abundance differences were examined using the Kruskal–Wallis test (*p* < 0.01) with the ALDEx2 package version 1.28.1 [20] within R version 4.3.3 [36], implemented in RStudio version 2023.06.1 [38].

The standard error for connectivity loss was calculated, incorporating variability with a threshold of 0.9. Node addition analyses were conducted in RStudio version 2023.06.1 [38]. Wilcoxon signed-rank tests were used to assess whether the mean size of the LCC and APL significantly differed from 0. *p* values were adjusted using the Benjamini–Hochberg procedure, and bootstrapping was applied in node removal analyses to estimate confidence intervals.

## Results

### Seasonal Variations in Diversity and Taxonomic Composition of *H. excavatum* Microbiome

**Fig. 1:**
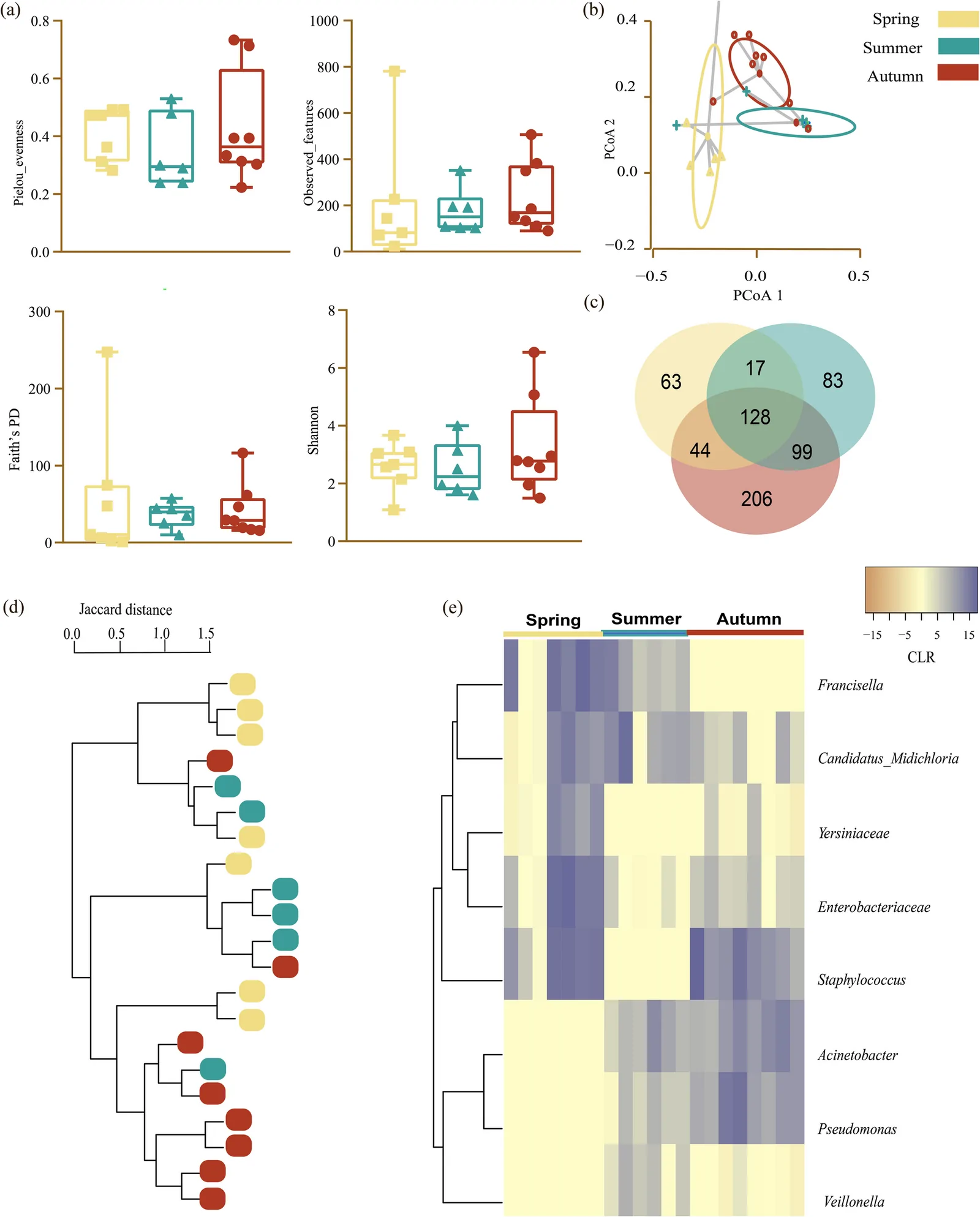
Comparison of the diversity of complex microbial communities within *H. excavatum* over seasons. **a** Comparison of alpha diversity among autumn, spring, and summer (Kruskal–Wallis test; no significant differences for *p* > 0.05). **b** Comparison of beta diversity with Bray–Curtis dissimilarity index among autumn, spring, and summer. Beta dispersion of the three sets of samples (global comparison) is shown. Small circles, crosses, and triangles represent samples, and ellipses represent the centroid position for each group. ANOVA indicated that the beta dispersion among the three sets of samples (three conditions) differed significantly (*p* ≤ 0.05). **c** Venn Diagram displaying the comparison of taxa composition in ticks collected at the three sampling times. Common and unique taxa between the conditions are shown. **d** Jaccard clustering of the tick samples collected in autumn, spring, and summer. The samples are represented by circles, and the groups by colors (see legend). **e** Differential taxonomic composition of microbial communities within *H. excavatum* across seasons. The taxa were clustered based on relative abundance (calculated as clr-transformed values). Each column represents the clr values for bacterial taxa per sample and per group. Each line represents bacterial taxa with significant changes between the datasets (ALDEx2, KW, *p* ≤ 0.05). Colors represent the clr values (ranging from − 15 to 15)

Distinct patterns were observed in the diversity and composition of microbial communities within *H. excavatum* ticks collected in spring, summer, and autumn. Alpha diversity metrics, including richness and evenness (Fig. 1a), showed no significant differences across the three seasons (Kruskal–Wallis test, *p* > 0.05). Beta diversity analysis, based on Bray–Curtis dissimilarity, revealed significant seasonal clustering of microbial communities (PERMANOVA: *R*^2^ = 0.23, *p* = 0.001; Fig. 1b). Pairwise comparisons showed that microbial compositions in spring differed significantly from both summer (*p*_adj_ = 0.0285) and autumn (*p*_adj_ = 0.0030), while summer and autumn communities were more similar (*p*_adj_ = 0.102). These findings suggest that while overall microbial richness remained stable, community composition was significantly shaped by seasonal variation.

The analysis of taxonomic composition (Fig. 1c) highlighted a group of microbial taxa shared across all seasons (Supplementary Table S1), accounting for the largest part of the microbial community. However, unique taxa were predominantly detected in autumn, reflecting potential environmental influences during this period. Jaccard clustering (Fig. 1d) provided additional support for seasonal differentiation, with samples clustering tightly within their respective seasons, underscoring the distinct microbial profiles associated with each sampling period. Furthermore, differential abundance analyses detected eight specific bacterial taxa with significant differences between seasons (Fig. 1e; Supplementary Table S2). For instance, *Francisella* and *Candidatus* Midichloria exhibited higher relative abundances in autumn compared to spring and summer. The families Yersiniaceae and Enterobacteriaceae were predominantly found in spring, while *Staphylococcus* was dominant in autumn and spring but not in summer. Conversely, *Acinetobacter*, *Pseudomonas*, and *Veillonella* were less abundant in spring.

### Seasonal Variation in *H. excavatum* Microbial Community Networks

**Fig. 2:**
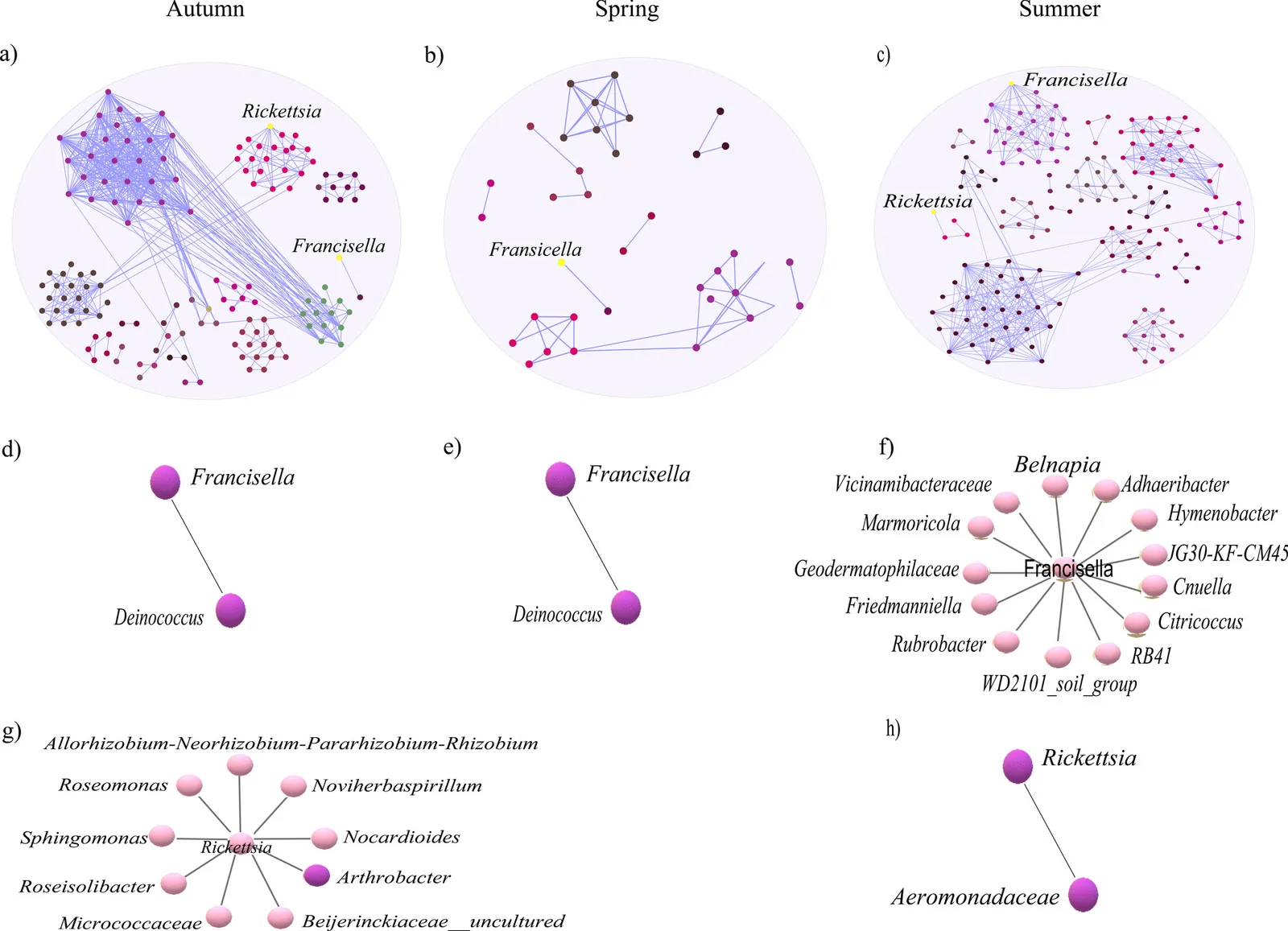
Seasonal variation and connectivity of *Francisella* and *Rickettsia*. **a**–**c** Co-occurrence networks of tick microbiota for (**a**) autumn, **b** spring, and (**c**) summer, where node colors indicate modularity classes (modules of co-occurring taxa), node size represents eigenvector centrality, and edge colors indicate strong positive correlations (blue). **d**–**h** Local connectivity and module composition of *Francisella* during (**d**) autumn, **e** spring, and (**f**) summer, and of *Rickettsia* during (**g**) autumn and (**h**) summer.

The seasonal variations of microbial community assembly in *H. excavatum* ticks were explored using co-occurrence networks constructed separately for autumn, spring, and summer, as illustrated in Fig. 2a–c. The autumn network showed intermediate complexity with 144 nodes and 538 edges (Fig. 2a; Table 1). The spring network was the simplest, with only 35 nodes and 51 edges (Fig. 2b; Table 1). The summer network was the most complex, with 169 nodes and 404 edges (Fig. 2c; Table 1), reflecting high microbial diversity and interactions. These networks provide insight into microbial interactions and community structures across seasons. Modularity varied across seasons, being highest in summer (0.77) and lowest in autumn (0.45) (Table 1), indicating more structured communities in warmer conditions. Other metrics, such as clustering coefficient and average degree, also highlighted distinct seasonal dynamics (Table 1). Seasonal differences significantly influenced the microbial networks in *H. excavatum*.

**Table 1:** Topological features of microbial networks with *Francisella* and *Rickettsia* present in each season

Network features	Autumn	Spring	Summer
No. of nodes	144	35	169
No. of edges	538	51	404
Positive interaction	538	51	404
Negative interaction	0	0	0
Modularity	0.45	0.7	0.77
Network diameter	9	5	8
Average degree	7.47	2.91	4.78
Weighted degree	6.01	2.52	3.83
Clustering coefficient	0.54	0.57	0.57
Connectivity	13	7	15

### Local Connectivity of *Francisella* and *Rickettsia*

Analyzing the local connectivity of *Francisella* (Fig. 2d–f) and *Rickettsia* (Fig. 2g, h) across seasons revealed notable differences in their associations and potential roles in microbial networks. In autumn, *Francisella* exhibited a specific relationship with *Deinococcus* (Fig. 2d), while *Rickettsia* exhibited multiple associations 9 taxa (Fig. 2g), highlighting distinct ecological roles for these taxa during this season. In spring, *Francisella* maintained its connection with *Deinococcus* (Fig. 2e), whereas *Rickettsia* was not detected in any sample, as confirmed by 16S rRNA sequencing data, emphasizing the seasonal absence of *Rickettsia* and the persistent influence of *Francisella*. In summer, *Francisella* displayed extensive connectivity, interacting with 14 taxa (Fig. 2f), compared to *Rickettsia*, which was related to 2 taxa (Fig. 2h). This broader connectivity of *Francisella* suggests a more dominant role in shaping microbial interactions during summer, potentially reflecting its ecological importance and adaptability across varying seasonal conditions.

### Impact of *Francisella* and *Rickettsia* on Microbial Community Assembly

**Fig. 3:**
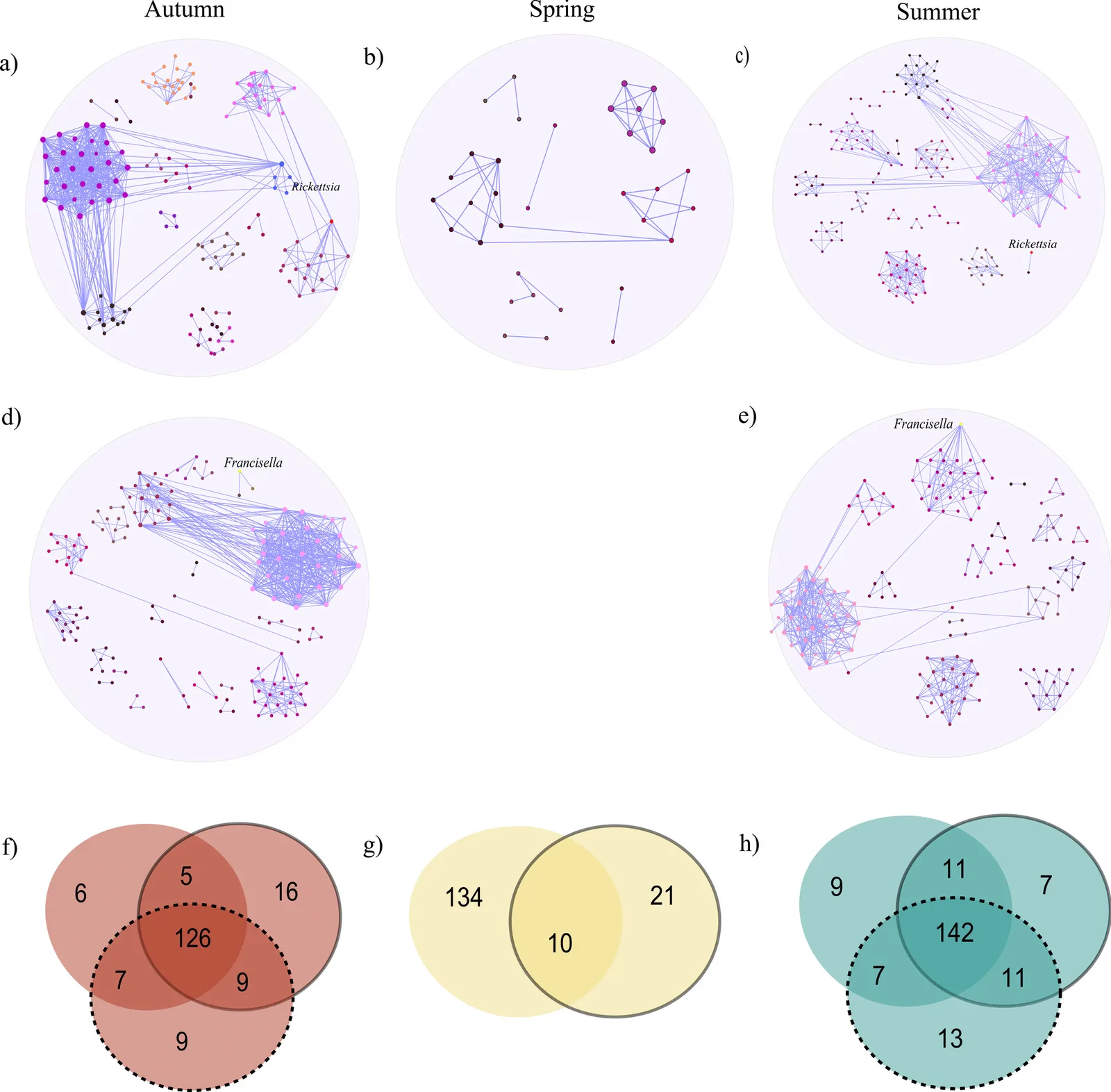
Impact of *Francisella* and *Rickettsia* removal on seasonal pathogen and module composition comparisons. **a**–**e** Sub-networks without *Francisella* in (**a**) autumn, **b** spring, and (**c**) summer, and without *Rickettsia* in (**d**) autumn and **e** summer. **f**–**h** Venn diagrams illustrate the seasonal comparison of microbial taxa compositions in autumn (**f**), spring (**g**), and summer (**h**), based on co-occurrence networks of *Hyalomma excavatum* ticks. Each diagram compares three network conditions for a given season: the original network including both *Francisella* and *Rickettsia* (N, shown with no contour), the network with *Francisella* removed (WoF, solid contour), and the network with *Rickettsia* removed (WoR, dotted contour). Overlaps represent shared microbial taxa among the networks, while non-overlapping areas reflect taxa unique to a specific condition. In panel (**g**), no dotted contour is shown because *Rickettsia* was absent in all spring samples, and thus a network without *Rickettsia* (WoR) could not be constructed

To evaluate the impact of *Francisella* and *Rickettsia* on the microbial community structure of *H. excavatum,* seasonal sub-networks were constructed excluding these taxa—without *Francisella* and without *Rickettsia* (Fig. 3a–e). When *Francisella* was removed, the number of nodes increased slightly across all seasons compared to the complete networks, with 151, 31, and 173 nodes in autumn, spring, and summer (Fig. 3a–c; Table 2), respectively. This suggests that *Francisella*’s dominance might influence the presence of other microbial taxa. The number of edges decreased marginally, with 533, 49, and 379 edges observed in autumn, spring, and summer, respectively (Table 2), when compared with the network with *Francisella* (Table 1). Positive interactions remained the sole type of correlation, and modularity values were consistent with the complete networks, showing a slightly structured microbial community, particularly in summer (0.75) (Tables 1 and 2).

**Table 2:** Topological features of microbial networks without the *Francisella* taxon in each season

Network features	Autumn	Spring	Summer
No. of nodes	151	31	173
No. of edges	533	49	379
Positive interaction	533	49	379
Negative interaction	0	0	0
Modularity	0.45	0.69	0.75
Network diameter	8	5	9
Average degree	7.06	3.16	4.38
Weighted degree	5.69	2.74	3.52
Clustering coefficient	0.53	0.66	0.54
Connectivity	19	6	18

Topological metrics such as network diameter and clustering coefficient showed nuanced differences. The clustering coefficient decreased in summer but increased in spring when *Francisella* was removed (Table 2), indicating changes in localized interaction density. Similarly, the network diameter expanded in autumn and summer, reflecting longer paths between taxa in the absence of *Francisella* (Table 2). These results suggest that *Francisella* plays a central role in shaping microbial network structure, particularly by maintaining connectivity and localized clustering in summer.

Excluding *Rickettsia* had a distinct impact on the microbial networks in autumn and summer, as it was absent from all samples in spring (Fig. 3d, e; Table 3). The number of nodes increased slightly to 156 and 171 in autumn and summer, respectively, while the number of edges decreased to 508 and 396, respectively (Table 3). Modularity values were similar to the networks without *Francisella*, with a structured and compartmentalized network observed in summer (Table 3). The clustering coefficient was highest in autumn, indicating tight interactions among remaining taxa, while the connectivity values dropped slightly compared to the networks with *Rickettsia* (Table 3).

**Table 3:** Topological features of microbial networks without the *Rickettsia* taxon in each season

Network features	Autumn	Summer
No. of nodes	156	171
No. of edges	508	396
Positive interaction	508	396
Negative interaction	0	0
Modularity	0.48	0.75
Network diameter	8	9
Average degree	6.51	4.36
Weighted degree	5.24	3.71
Clustering coefficient	0.58	0.56
Connectivity	21	14

Comparing the sub-networks without *Francisella* or *Rickettsia* highlights their differential roles in the microbial ecology of *H. excavatum*. While both taxa contribute significantly to network structure and interactions, *Francisella* appears to have a stronger effect on network density and clustering, particularly in summer. *Rickettsia*, on the other hand, influences connectivity and overall interaction dynamics.

### Comparison of Community Compositions Across Seasons With and Without Francisella and Rickettsia

The microbial communities in *H. excavatum* ticks were analyzed using Venn diagrams to identify taxa shared across and exclusively present in one of the three networks—network with *Francisella* and *Rickettsia* (N), network without *Francisella* (WoF), and network without *Rickettsia* (WoR)—across autumn, spring, and summer (Fig. 3f–h; Supplementary Table S3). Here, “unique” refers to taxa that were exclusively detected in one network and completely absent in the others. In autumn (Fig. 3f), 126 taxa formed a stable community shared across all networks, while the removal of *Francisella* or *Rickettsia* revealed distinct patterns. WoF included 16 unique taxa, and WoR revealed nine, demonstrating their individual roles in the microbial network (Fig. 3f). In spring (Fig. 3g), *Francisella*’s dominance profoundly influenced the community. Removing *Francisella* exposed 21 unique taxa in WoF that were previously overshadowed, while only 10 taxa were shared between N and WoF. In summer (Fig. 3h), 142 taxa were consistently shared across all networks, indicating a resilient core. However, removing *Francisella* or *Rickettsia* revealed 7 and 13 unique taxa in WoF and WoR (Fig. 3h), respectively, emphasizing their contributions to the microbial structure. These findings reveal a dynamic interplay between core and unique taxa, underscoring the significant roles of *Francisella* and *Rickettsia* in shaping the tick microbiome across seasons.

### The Robustness Comparison of Microbial Networks Under Various Node Addition and Removal Scenarios Across Seasons

**Fig. 4:**
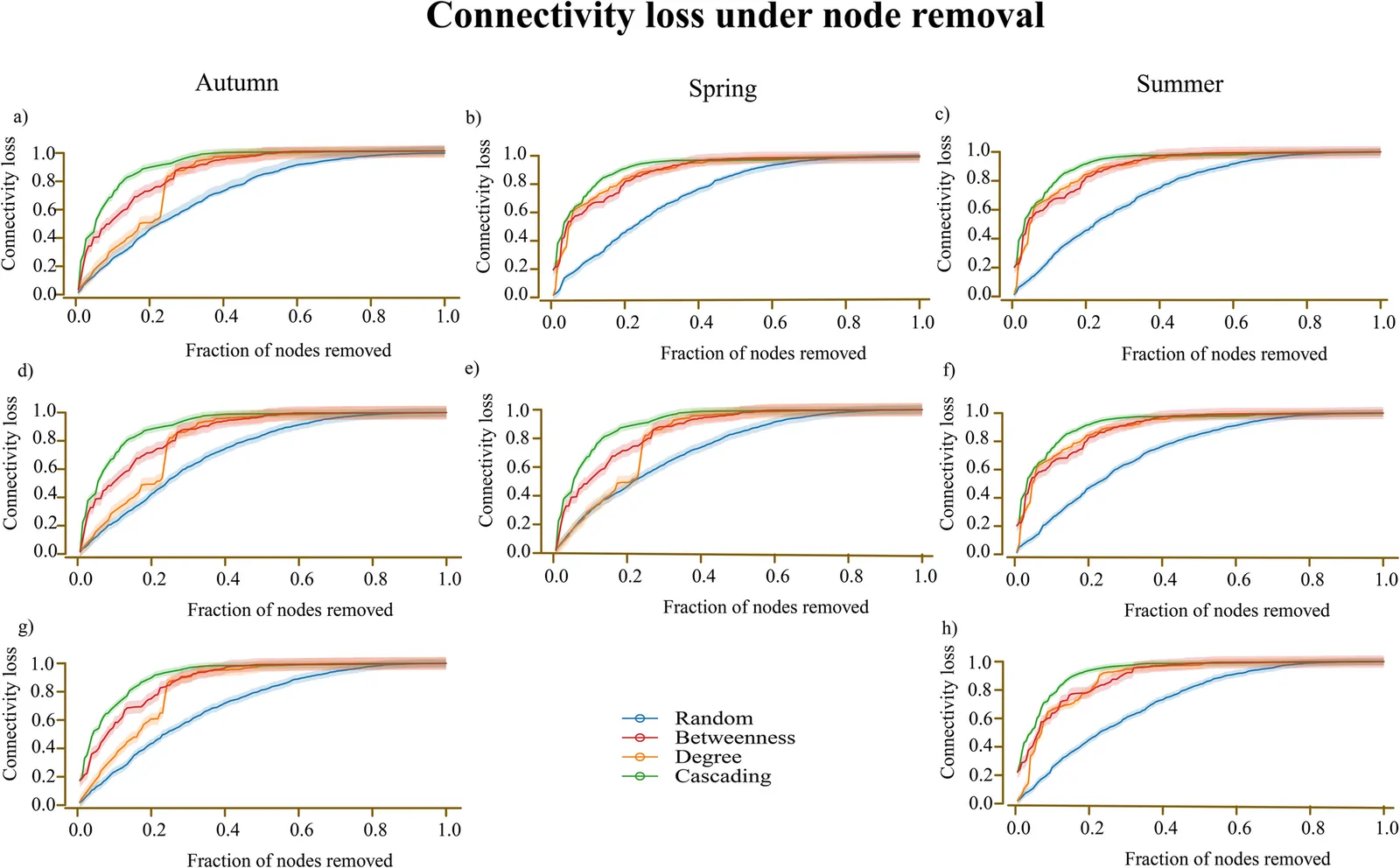
Seasonal effects of *Francisella* and *Rickettsia* on network robustness. Connectivity loss under node addition and removal scenarios is depicted for various attack strategies—betweenness (red), cascading (green), degree (orange), and random (blue)—in networks with *Francisella* and *Rickettsia* presence in (**a**) autumn, **b** spring, and (**c**) summer; with *Francisella* absent in (**d**) autumn, **e** spring, and (**f**) summer; and *Rickettsia* absence in (**g**) autumn and (**h**) summer

Network robustness varies across seasons and is influenced by the presence of *Francisella* and *Rickettsia*. Cascading failures (green) and betweenness-based attacks (red) emerge as the most disruptive, rapidly reducing connectivity. In the autumn network, where both *Francisella* and *Rickettsia* are present (Fig. 4a), connectivity drops by nearly 80% when only 20% of nodes are removed under cascading failure, whereas random attacks (blue) lead to a more gradual decline, with less than 40% connectivity loss at the same removal fraction. A similar pattern is observed in Fig. 4b, though the gap between cascading and other targeted attacks is smaller, indicating variations in network resilience. Seasonal differences further influence robustness, with the summer network (Fig. 4c) exhibiting greater stability, as connectivity remains higher across all attack strategies compared to autumn and spring. The absence of *Francisella* (Fig. 4d–f) increases network vulnerability, particularly under cascading and degree-based attacks, while the removal of *Rickettsia* (Fig. 4g–h) similarly reduces structural stability. For example, in the autumn network without *Rickettsia* (Fig. 4g), betweenness and cascading attacks resulted in over 90% connectivity loss when 30% of nodes were removed. Network robustness varied across seasons and was influenced by the presence of *Francisella* and *Rickettsia*. The results indicated that cascading failures (green) and betweenness-based attacks (red) were the most disruptive, causing rapid connectivity loss.

**Fig. 5:**
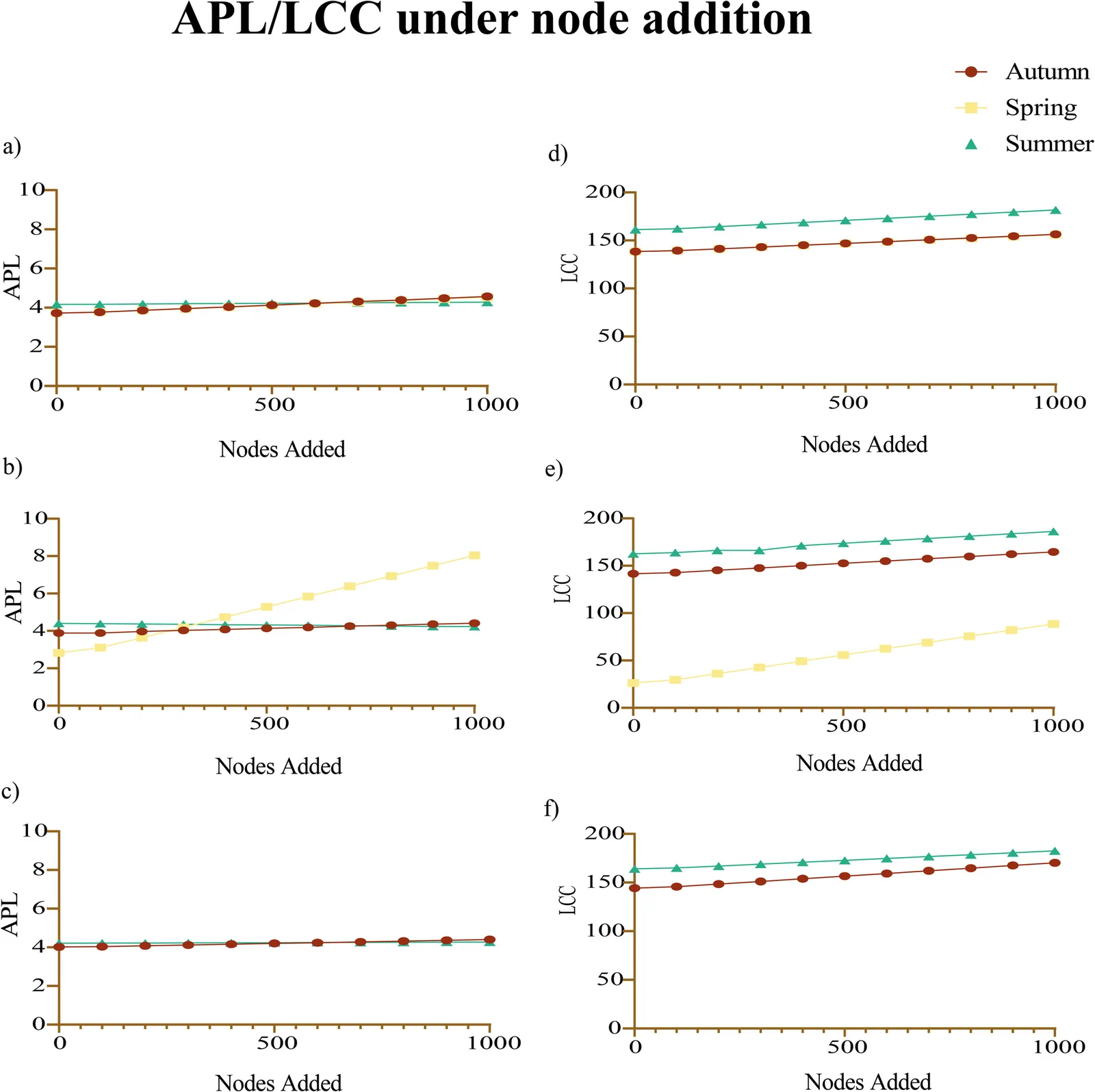
Seasonal effects of *Francisella* and *Rickettsia* on network structure. Average Path Length (APL) values are shown for networks with *Francisella* and *Rickettsia* present (**a**), *Francisella* absent (**b**), and *Rickettsia* absent (**c**) across seasons. Largest Connected Component (LCC) values are compared across seasons for networks with *Francisella* and *Rickettsia* present (**d**), *Francisella* absent (**e**), and *Rickettsia* absent (**f**)

The comparison of predicted APL values across autumn, spring, and summer microbial networks (Fig. 5a) demonstrates clear seasonal variations in response to node addition. In all three seasons, APL gradually increases as nodes are added, indicating a consistent structural trend. The autumn network (3.72 to 4.62) closely parallels the spring network (3.73 to 4.57), while the summer network starts at a higher APL (4.17) and increases modestly to 4.29, suggesting a more compact baseline structure. Despite overall similarity, autumn and spring diverge in later stages, with autumn reaching a higher final APL, indicating slightly more fragmentation. The summer network maintains the lowest range of APL values, highlighting greater inherent connectivity and robustness under node addition (Fig. 4i).

The removal of *Francisella* (Fig. 5b) revealed notable changes in network robustness, particularly in spring, where its absence significantly increased APL. In autumn, *Francisella* removal increased APL from 3.89 to 4.44, though the effect was weaker than that of *Rickettsia* removal. In summer, *Francisella* removal caused minimal changes (4.18 to 4.25), indicating a limited impact compared to autumn.

The removal of *Rickettsia* (Fig. 5c) had varying effects across seasons. In autumn, APL increased more significantly than with *Francisella* removal, suggesting a greater impact on network fragmentation. In spring, *Rickettsia* removal resulted in minimal changes compared to *Francisella* removal, highlighting differences in their roles across seasons. The summer network exhibited a slight increase in APL, reinforcing the idea that *Rickettsia* plays a more limited stabilizing role in this season.

The LCC analysis (Fig. 5d) further highlights the season-specific roles of *Francisella* and *Rickettsia*. In autumn, both bacteria maintained LCC values between 138.42 and 157.27. When *Francisella* was removed (Fig. 5e), LCC values increased (141.61 to 165.84), indicating a destabilizing effect, while *Rickettsia* removal (Fig. 5f) led to even higher LCC values (144.30 to 171.62), suggesting it also weakens network robustness. In spring, *Francisella* removal caused a significant drop in LCC (26.22 to 91.90), underscoring its essential role in maintaining network connectivity, whereas in summer, *Francisella* removal slightly increased LCC (162.61 to 187.45), suggesting a minor destabilizing effect, while *Rickettsia* removal further raised LCC (164.03 to 183.52), indicating a limited stabilizing role in this season.

## Discussion

The microbiome of *Hyalomma excavatum* ticks is subject to seasonal variation, influencing microbial diversity, pathogen prevalence, and interspecies interactions. This study highlights how key microbial taxa, such as *Francisella* and *Rickettsia*, play significant roles in shaping the tick microbiome, with their relative abundance and microbial network connectivity fluctuating across different seasons. These findings align with previous research on other tick species, suggesting that microbiome dynamics are driven by environmental factors, including temperature and humidity [25]. However, by focusing on *H. excavatum*, a relatively underexplored vector compared to well-studied ticks like *Ixodes*, this study provides novel insights into how seasonal shifts shape its microbial community and pathogen associations. These findings contribute to a better understanding of *H. excavatum*’s potential role in pathogen transmission under changing ecological conditions.

Environmental factors, such as temperature, are known to be major drivers of tick microbiome structure [41]. Our results show distinct seasonal shifts in microbial communities, with *Francisella* and *Candidatus* Midichloria being more abundant in autumn, whereas Yersiniaceae and Enterobacteriaceae were predominant in spring. These seasonal fluctuations are consistent with findings in *Ixodes scapularis*, where bacterial diversity was significantly affected by temperature [41]. However, there are discrepancies across studies regarding the impact of high temperatures on bacterial diversity in ticks.

In *I. scapularis*, high temperatures reduced microbial diversity and weakened microbial network connectivity [46]. In contrast, in *Ixodes ricinus*, microbial interactions became more complex in warmer conditions, suggesting that higher temperatures might facilitate co-occurrence of microbial taxa rather than cause diversity loss [25]. On the other hand, the *H. dromedarii* study in the UAE reported that microbial diversity remained stable despite seasonal variations, indicating a more resilient microbiome [31]. Our study on *H. excavatum* aligns more closely with *I. ricinus*, as we observed higher microbial network complexity in summer, indicating that warmer conditions may enhance microbial interactions rather than reduce diversity.

Several factors could explain these discrepancies. Species-specific adaptations may play a critical role, as *H. dromedarii* is adapted to desert conditions, potentially harboring a microbiome that is more resistant to extreme temperature shifts. Conversely, *I. scapularis*, which inhabits temperate regions, may experience greater microbiome disruption under heat stress. Another possible explanation is differences in experimental conditions. The study on *I. scapularis* was conducted under controlled laboratory settings [41], where heat stress was imposed artificially, whereas the studies on *Hyalomma* [31] species and *I. ricinus* [25] used field-collected ticks, allowing for natural environmental buffering, including potential microbial acquisition from the habitat.

Recent research by Abdelali et al. [1], using the same *H. excavatum* tick samples analyzed here, provides valuable insight into pathogen–pathogen interactions. Their study revealed that certain pathogen guilds exhibit cooperative associations, such as *Rickettsia slovaca* and *Rickettsia conorii*, while others, such as *Anaplasma phagocytophilum* and *Francisella tularensis*, may engage in competitive exclusion. In the present network analysis, this specific negative association was not observed, likely due to differences in detection thresholds or seasonal filtering. It is important to note that the current study was based on presence/absence data derived from 16S rRNA gene sequencing, not on quantitative pathogen load, which may influence the resolution of inferred interactions. These findings suggest that tick microbiomes may influence pathogen persistence and co-occurrence patterns, a hypothesis supported by our results showing the pivotal role of *Francisella* and *Rickettsia* in microbial networks.

Abdelali et al. [1] also observed seasonal differences in pathogen prevalence, with female ticks exhibiting higher infection rates than males. This aligns with our microbiome findings, where seasonality influenced microbial community structure and complexity. The seasonal co-occurrence of certain bacteria and pathogens suggests that environmental factors regulate both microbiota and pathogen load, shaping tick-borne disease transmission dynamics.

The positive and negative pathogen–pathogen interactions reported by Abdelali et al. [1] could be mediated by microbiome composition. For instance, the negative association between *A. phagocytophilum* and *F. tularensis* suggests that *Francisella* may play a role in pathogen exclusion. Our study found that *Francisella* is highly connected, which could be influencing the tick’s pathogen load by either outcompeting certain pathogens or indirectly altering microbial community dynamics. These results reinforce the hypothesis that the tick microbiome may act as a selective filter for pathogen colonization [29, 45].

The stability of microbial networks may be influenced by taxa that occupy central or structurally important positions within the community. In *Hyalomma excavatum*, *Francisella* and *Rickettsia* consistently maintained microbial balance across seasons, acting as potential stabilizers within the network due to their strong connectivity and persistence. Similarly, in *Ixodes scapularis*, certain thermostable bacteria such as *Pseudomonas*, *Ralstonia*, *Acinetobacter*, and *Bradyrhizobium* were identified as key contributors to microbiome stability under heat stress [46]. However, while these bacteria were lost at higher temperatures in *I. scapularis*, microbial interactions in *H. excavatum* appeared to be enhanced rather than diminished, as evidenced by *Francisella* being connected to 13 taxa in the summer network, compared to only one taxon in both spring and autumn. This contrast suggests that functional redundancy, where different microbial taxa can fulfill similar ecological roles, may buffer some tick species against environmental fluctuations, whereas others remain more sensitive to thermal stress.

The seasonal modulation of microbial networks has direct implications for tick-borne disease transmission. Increased *Rickettsia* abundance in specific seasons may enhance pathogen transmission, similar to findings in *I. ricinus*, where *Borrelia* and *Anaplasma* disrupted microbial interactions and increased disease risk [25]. Additionally, the study on *I. scapularis* [41] highlights that heat stress reduces microbial diversity but preserves key metabolic pathways [46], suggesting that keystone bacteria buffer environmental fluctuations. This functional stability may contribute to the persistence of tick-borne pathogens across different seasons, reinforcing the importance of microbiome monitoring in tick control strategies.

A crucial aspect of microbial ecology is whether observations in controlled environments translate to natural conditions. The *I. scapularis* study found that lab-reared and field-collected ticks harbored similar keystone bacteria [46], suggesting that laboratory experiments can accurately reflect microbial dynamics in natural tick populations. Our results align with this, indicating that seasonal shifts in *H. excavatum* microbiomes observed in the field are not random, but rather follow predictable environmental patterns. Future research should explore how microbiome stability varies across tick species under controlled and field conditions, focusing on the functional implications of microbial shifts for vector competence and pathogen transmission.

Despite the valuable insights provided by this study, several limitations should be acknowledged. The sample size was limited to 21 engorged female *H. excavatum* ticks, which may affect the statistical power and generalizability of the findings. Although all ticks were collected during the early stage of engorgement and no host DNA contamination was detected using the Decontam tool, individual variation in host immune status and feeding duration could still influence the microbiome profiles. Furthermore, the absence of unfed or male ticks limits our ability to assess sex- or feeding-related microbial differences. These constraints highlight the need for future studies with larger and more diverse sample sets under controlled experimental conditions.

## Conclusions

This study provides the first characterization of the seasonal variation of the *H. excavatum* microbiome in Algeria, revealing significant variations in microbial diversity, composition, and network stability. The findings highlight the role of FLEs in structuring microbial interactions and influencing pathogen presence, particularly in relation to *Rickettsia*. Seasonal shifts impact microbiome resilience, with potential implications for pathogen transmission dynamics. Understanding these microbial patterns enhances our knowledge of tick-borne disease ecology and supports the development of targeted vector control strategies. Future research should explore the functional consequences of these microbial interactions and their broader epidemiological significance.

## Supplementary Information

Below is the link to the electronic supplementary material.

Supplementary Material 1 (DOCX 28.2 KB)

Supplementary Material 2 (DOCX 16.1 KB)

Supplementary Material 3 (DOCX 27.2 KB)

## Data Availability

The raw sequencing data used to support the findings of this study have been deposited in the NCBI Sequence Read Archive (SRA) under project number [PRJNA1214082].
