# Supplementary material for: Seasonal Variations in the Microbiome of Hyalomma excavatum Ticks in Algeria
Source: Microb Ecol. 2025 Sep 30;88(1):96. doi: 10.1007/s00248-025-02597-y (PMC12484380; doi:10.1007/s00248-025-02597-y)
Supplement: Supplementary file 1 — Supplementary Material 1 (DOCX 28.2 KB) [file 248_2025_2597_MOESM1_ESM.docx]

**Supplementary Table S1:** Core microbial taxa shared across seasons

| Names | Total | Elements |
| --- | --- | --- |
| Autumn Spring Summer | 128 | g__Segetibacter |
|  |  | g__Mannheimia |
|  |  | f__Gemmatimonadaceae__uncultured |
|  |  | g__Moraxella |
|  |  | g__Aeromonas |
|  |  | f__Geodermatophilaceae |
|  |  | f__Pasteurellaceae |
|  |  | g__Francisella |
|  |  | f__Beijerinckiaceae__uncultured |
|  |  | g__Geminicoccus |
|  |  | o__Bacillales |
|  |  | g__Methanobrevibacter |
|  |  | g__Bibersteinia |
|  |  | g__Selenomonas |
|  |  | g__Aquipuribacter |
|  |  | g__UCG-005 |
|  |  | g__Rubrobacter |
|  |  | g__Christensenellaceae_R-7_group |
|  |  | g__Brachybacterium |
|  |  | g__Blastococcus |
|  |  | g__PMMR1 |
|  |  | g__Deinococcus |
|  |  | g__Haemophilus |
|  |  | g__Sphingobacterium |
|  |  | g__Pontibacter |
|  |  | g__Corynebacterium |
|  |  | g__Iamia |
|  |  | g__Clostridium_sensu_stricto_1 |
|  |  | f__Microbacteriaceae |
|  |  | f__Micrococcaceae |
|  |  | g__Kocuria |
|  |  | g__Streptomyces |
|  |  | f__Carnobacteriaceae |
|  |  | g__Cnuella |
|  |  | f__Comamonadaceae |
|  |  | g__Arthrobacter |
|  |  | g__JG30-KF-CM66 |
|  |  | g__Pedobacter |
|  |  | f__Planococcaceae |
|  |  | g__Paracoccus |
|  |  | g__Turicibacter |
|  |  | f__Kineosporiaceae |
|  |  | g__Noviherbaspirillum |
|  |  | g__Lysinibacillus |
|  |  | g__Actinomadura |
|  |  | f__Enterobacteriaceae |
|  |  | g__Atopostipes |
|  |  | g__Solirubrobacter |
|  |  | g__Skermanella |
|  |  | g__Actinomycetospora |
|  |  | g__Cutibacterium |
|  |  | g__Candidatus_Alysiosphaera |
|  |  | g__Sphingomonas |
|  |  | g__Rickettsia |
|  |  | g__Truepera |
|  |  | f__Intrasporangiaceae |
|  |  | g__WD2101_soil_group |
|  |  | g__Bryobacter |
|  |  | g__JG30-KF-CM45 |
|  |  | g__Massilia |
|  |  | g__Bacillus |
|  |  | g__Candidatus_Midichloria |
|  |  | g__Stenotrophomonas |
|  |  | g__Pseudonocardia |
|  |  | g__Lachnospiraceae_NK3A20_group |
|  |  | g__Pseudomonas |
|  |  | g__Geodermatophilus |
|  |  | g__Bergeyella |
|  |  | g__Exiguobacterium |
|  |  | g__Mycobacterium |
|  |  | f__Beijerinckiaceae |
|  |  | g__Hymenobacter |
|  |  | o__Vicinamibacterales__uncultured |
|  |  | g__Brevibacterium |
|  |  | g__TM7a |
|  |  | c__Acidimicrobiia__uncultured |
|  |  | g__Microvirga |
|  |  | g__Adhaeribacter |
|  |  | g__Qipengyuania |
|  |  | g__Saccharimonadales |
|  |  | g__Streptococcus |
|  |  | g__Devosia |
|  |  | g__Chryseobacterium |
|  |  | g__Escherichia-Shigella |
|  |  | g__Mitochondria |
|  |  | g__Citricoccus |
|  |  | f__Acetobacteraceae |
|  |  | f__Neisseriaceae__uncultured |
|  |  | g__Brevundimonas |
|  |  | g__Alloprevotella |
|  |  | o__Frankiales__uncultured |
|  |  | f__Isosphaeraceae__uncultured |
|  |  | g__Alysiella |
|  |  | g__Lactobacillus |
|  |  | g__Friedmanniella |
|  |  | g__Allorhizobium-Neorhizobium-Pararhizobium-Rhizobium |
|  |  | g__[Eubacterium]_coprostanoligenes_group |
|  |  | g__Modestobacter |
|  |  | f__Rhodobacteraceae |
|  |  | d__Bacteria |
|  |  | g__Staphylococcus |
|  |  | g__Mesorhizobium |
|  |  | f__Neisseriaceae |
|  |  | g__Nocardioides |
|  |  | g__Gemmatimonas |
|  |  | g__Cellulomonas |
|  |  | g__Mogibacterium |
|  |  | g__Georgenia |
|  |  | g__Methylobacterium-Methylorubrum |
|  |  | g__Ferruginibacter |
|  |  | g__Roseisolibacter |
|  |  | g__RB41 |
|  |  | f__Sphingomonadaceae |
|  |  | g__Kineococcus |
|  |  | g__Flavobacterium |
|  |  | g__Paenibacillus |
|  |  | g__Dietzia |
|  |  | g__Romboutsia |
|  |  | g__Rubellimicrobium |
|  |  | g__Craurococcus-Caldovatus |
|  |  | g__Oceanobacillus |
|  |  | g__Sporosarcina |
|  |  | g__Ornithinimicrobium |
|  |  | g__Prevotella |
|  |  | g__Acinetobacter |
|  |  | g__Aerococcus |
|  |  | g__Longimicrobium |
|  |  | g__Lawsonella |
| Autumn Spring | 45 |  |
|  |  | g__Flaviaesturariibacter |
|  |  | o__Enterobacterales |
|  |  | g__Pantoea |
|  |  | g__Trichococcus |
|  |  | g__Luteimonas |
|  |  | g__Agrococcus |
|  |  | g__Aeromicrobium |
|  |  | g__Edaphobaculum |
|  |  | g__Paenarthrobacter |
|  |  | f__Rhizobiaceae |
|  |  | o__Rhizobiales__uncultured |
|  |  | g__Shewanella |
|  |  | g__Clostridioides |
|  |  | g__Lactococcus |
|  |  | f__Caldilineaceae__uncultured |
|  |  | g__Altererythrobacter |
|  |  | g__Olsenella |
|  |  | f__Myxococcaceae__uncultured |
|  |  | g__Limnobacter |
|  |  | f__Caulobacteraceae__uncultured |
|  |  | g__Glutamicibacter |
|  |  | g__Longimicrobiaceae |
|  |  | g__Bdellovibrio |
|  |  | g__Methanosphaera |
|  |  | o__Gaiellales |
|  |  | g__Muribaculaceae |
|  |  | g__Enteractinococcus |
|  |  | g__Paeniclostridium |
|  |  | g__Lysobacter |
|  |  | g__Antricoccus |
|  |  | g__Rikenellaceae_RC9_gut_group |
|  |  | g__Oligoflexus |
|  |  | f__Cyclobacteriaceae |
|  |  | g__EPR3968-O8a-Bc78 |
|  |  | g__WPS-2 |
|  |  | g__Macrococcus |
|  |  | g__Jeotgalicoccus |
|  |  | g__F082 |
|  |  | f__Yersiniaceae |
|  |  | g__Alistipes |
|  |  | g__Anaerosporobacter |
|  |  | g__Mailhella |
|  |  | o__Coriobacteriales |
|  |  | o__Lactobacillales |
| Autumn Summer | 99 | g__Subgroup_7 |
|  |  | g__Vulcaniibacterium |
|  |  | g__Cloacibacterium |
|  |  | g__Veillonella |
|  |  | g__Candidatus_Saccharimonas |
|  |  | g__67-14 |
|  |  | f__Ruminococcaceae__uncultured |
|  |  | f__Isosphaeraceae |
|  |  | g__Rheinheimera |
|  |  | g__Anaerococcus |
|  |  | g__Cardiobacterium |
|  |  | g__Quadrisphaera |
|  |  | g__Absconditabacteriales_(SR1) |
|  |  | g__Novosphingobium |
|  |  | g__Fusobacterium |
|  |  | f__Bacillaceae |
|  |  | g__Enhydrobacter |
|  |  | g__Nitrospira |
|  |  | g__RF39 |
|  |  | f__Gemmataceae__uncultured |
|  |  | g__Vogesella |
|  |  | g__Gracilibacteria |
|  |  | g__TK10 |
|  |  | g__Family_XIII_AD3011_group |
|  |  | g__Rhodococcus |
|  |  | f__Sporichthyaceae__uncultured |
|  |  | f__Dermabacteraceae |
|  |  | g__Syntrophococcus |
|  |  | g__Bifidobacterium |
|  |  | o__Gaiellales__uncultured |
|  |  | g__Marmoricola |
|  |  | f__Microtrichaceae__uncultured |
|  |  | g__Saccharomonospora |
|  |  | g__Aquabacterium |
|  |  | g__KD4-96 |
|  |  | g__Riemerella |
|  |  | g__S0134_terrestrial_group |
|  |  | g__Xanthomonas |
|  |  | f__Micromonosporaceae |
|  |  | g__YC-ZSS-LKJ147 |
|  |  | p__Armatimonadota__uncultured |
|  |  | g__Lautropia |
|  |  | g__S085 |
|  |  | g__Vicinamibacteraceae |
|  |  | g__Conexibacter |
|  |  | g__Helcococcus |
|  |  | g__Woeseia |
|  |  | g__Rothia |
|  |  | g__Clostridia_UCG-014 |
|  |  | g__Bradyrhizobium |
|  |  | g__Aureimonas |
|  |  | g__Gemella |
|  |  | f__Carnobacteriaceae__uncultured |
|  |  | g__Rubritepida |
|  |  | f__Lachnospiraceae |
|  |  | g__MB-A2-108 |
|  |  | g__Schlegelella |
|  |  | g__Parviterribacter |
|  |  | o__Planctomycetales__uncultured |
|  |  | g__Coprococcus |
|  |  | f__Blastocatellaceae__uncultured |
|  |  | o__Microtrichales__uncultured |
|  |  | g__Roseomonas |
|  |  | f__Lachnospiraceae__uncultured |
|  |  | g__NK4A214_group |
|  |  | f__Ilumatobacteraceae__uncultured |
|  |  | f__Blastocatellaceae |
|  |  | g__Nocardiopsis |
|  |  | g__Bosea |
|  |  | g__Candidatus_Nitrososphaera |
|  |  | g__Actinomyces |
|  |  | g__Pir4_lineage |
|  |  | o__Frankiales |
|  |  | g__Anoxybacillus |
|  |  | g__AKIW781 |
|  |  | g__Patulibacter |
|  |  | g__Candidatus_Protochlamydia |
|  |  | f__Thermomonosporaceae |
|  |  | g__Flavisolibacter |
|  |  | g__Hydrogenophilus |
|  |  | f__Rhodobacteraceae__uncultured |
|  |  | g__Neisseria |
|  |  | g__Micrococcus |
|  |  | c__Acidimicrobiia |
|  |  | f__Ruminococcaceae |
|  |  | g__Leptotrichia |
|  |  | g__Gaiella |
|  |  | f__Aerococcaceae__uncultured |
|  |  | c__Clostridia |
|  |  | g__Chthoniobacter |
|  |  | g__Belnapia |
|  |  | f__Erysipelotrichaceae__uncultured |
|  |  | g__Hydrogenophaga |
|  |  | g__Psychroglaciecola |
|  |  | g__Porphyromonas |
|  |  | g__Geobacillus |
|  |  | g__Rhodocytophaga |
|  |  | g__Trueperella |
|  |  | g__Enterococcus |
| Spring Summer | 17 | f__Microscillaceae__uncultured |
|  |  | g__Steroidobacter |
|  |  | g__Psychrobacter |
|  |  | g__Acidiphilium |
|  |  | g__Empedobacter |
|  |  | g__Amaricoccus |
|  |  | g__Leuconostoc |
|  |  | g__Actinophytocola |
|  |  | f__Aerococcaceae |
|  |  | g__Nitrososphaeraceae |
|  |  | g__Spirosoma |
|  |  | g__Thermomonas |
|  |  | g__Candidatus_Nitrocosmicus |
|  |  | g__Virgibacillus |
|  |  | g__Rubrivirga |
|  |  | g__Tundrisphaera |
|  |  | g__Chloroplast |
| Autumn | 206 | g__Hyphomicrobium |
|  |  | g__Klenkia |
|  |  | g__Saccharimonadaceae |
|  |  | g__Pseudolabrys |
|  |  | g__Haliangium |
|  |  | g__Blfdi19 |
|  |  | g__Sphingopyxis |
|  |  | g__Devosiaceae |
|  |  | g__Candidatus_Peribacteria |
|  |  | g__P2-11E |
|  |  | f__Geobacteraceae |
|  |  | g__Stenotrophobacter |
|  |  | f__Caloramatoraceae |
|  |  | g__Gastranaerophilales |
|  |  | g__Asanoa |
|  |  | g__Alkanindiges |
|  |  | g__Monoglobus |
|  |  | g__Curvibacter |
|  |  | g__Caldicoprobacter |
|  |  | g__Anaeromyxobacter |
|  |  | g__MVP-15 |
|  |  | g__Sericytochromatia |
|  |  | g__[Ruminococcus]_gnavus_group |
|  |  | g__Peptoniphilus |
|  |  | g__Suttonella |
|  |  | g__Phyllobacterium |
|  |  | g__Negativicoccus |
|  |  | f__Cellulomonadaceae |
|  |  | g__CL500-29_marine_group |
|  |  | g__Flavitalea |
|  |  | g__Cryptosporangium |
|  |  | g__Rhodopseudomonas |
|  |  | g__Defluviicoccus |
|  |  | f__Geminicoccaceae__uncultured |
|  |  | o__Oscillospirales |
|  |  | g__Phreatobacter |
|  |  | g__Slackia |
|  |  | f__Rhodocyclaceae |
|  |  | g__Desemzia |
|  |  | f__Anaerolineaceae__uncultured |
|  |  | g__Alkaliphilus |
|  |  | g__Parasutterella |
|  |  | o__Azospirillales__uncultured |
|  |  | g__Caldibacillus |
|  |  | f__Clostridiaceae |
|  |  | g__Phenylobacterium |
|  |  | f__Solirubrobacteraceae |
|  |  | g__Neochlamydia |
|  |  | g__Terrisporobacter |
|  |  | g__[Eubacterium]_ventriosum_group |
|  |  | g__Peredibacter |
|  |  | o__Rhizobiales |
|  |  | f__Ilumatobacteraceae |
|  |  | g__Dactylosporangium |
|  |  | g__Aequorivita |
|  |  | g__Candidatus_Soleaferrea |
|  |  | f__Peptostreptococcaceae |
|  |  | o__Acidobacteriales |
|  |  | g__0319-6G20 |
|  |  | g__TRA3-20 |
|  |  | g__Tolumonas |
|  |  | g__BD2-11_terrestrial_group |
|  |  | g__Proteocatella |
|  |  | f__Solirubrobacteraceae__uncultured |
|  |  | g__Vermiphilaceae |
|  |  | f__Nocardioidaceae |
|  |  | g__Thermonema |
|  |  | g__Glycomyces |
|  |  | f__Pseudonocardiaceae |
|  |  | g__UCG-010 |
|  |  | f__Prolixibacteraceae__uncultured |
|  |  | f__Weeksellaceae |
|  |  | g__DNF00809 |
|  |  | g__Neo-b11 |
|  |  | g__Halomonas |
|  |  | g__Planifilum |
|  |  | g__Promicromonospora |
|  |  | f__Oxalobacteraceae |
|  |  | o__Xanthomonadales__uncultured |
|  |  | g__Muribaculum |
|  |  | g__Xanthobacter |
|  |  | g__Microbacterium |
|  |  | g__Caenimonas |
|  |  | g__Rufibacter |
|  |  | o__Solirubrobacterales |
|  |  | g__Fervidobacterium |
|  |  | g__Ochrobactrum |
|  |  | g__Facklamia |
|  |  | g__Marisediminicola |
|  |  | p__Patescibacteria__uncultured |
|  |  | f__Rikenellaceae |
|  |  | g__Abditibacterium |
|  |  | g__Thermoactinomyces |
|  |  | g__Amb-16S-1323 |
|  |  | g__Ellin6067 |
|  |  | g__Duganella |
|  |  | g__Flexilinea |
|  |  | f__Cellvibrionaceae__uncultured |
|  |  | f__Hyphomicrobiaceae |
|  |  | g__Asticcacaulis |
|  |  | g__[Ruminococcus]_gauvreauii_group |
|  |  | g__Dielma |
|  |  | g__P3OB-42 |
|  |  | g__Sphaerisporangium |
|  |  | g__Bordetella |
|  |  | f__Pseudomonadaceae |
|  |  | f__Hungateiclostridiaceae__uncultured |
|  |  | o__Elsterales__uncultured |
|  |  | f__Moraxellaceae__uncultured |
|  |  | g__Treponema |
|  |  | g__Sphingobium |
|  |  | f__Selenomonadaceae |
|  |  | g__Legionella |
|  |  | g__Elev-16S-1166 |
|  |  | g__Solibacillus |
|  |  | f__Xanthomonadaceae |
|  |  | g__Haloactinopolyspora |
|  |  | f__Acetobacteraceae__uncultured |
|  |  | g__Tepidimonas |
|  |  | g__Ramlibacter |
|  |  | g__Opitutus |
|  |  | f__Rubinisphaeraceae |
|  |  | g__SC-I-84 |
|  |  | g__Caulobacter |
|  |  | g__Bacteroides |
|  |  | g__Gemmata |
|  |  | f__Oscillospiraceae |
|  |  | g__Propionibacterium |
|  |  | g__Erysipelotrichaceae_UCG-009 |
|  |  | g__Fonticella |
|  |  | g__Candidatus_Entotheonella |
|  |  | g__BIrii41 |
|  |  | g__Armatimonadales |
|  |  | o__Saccharimonadales |
|  |  | g__Niveibacterium |
|  |  | g__Porphyrobacter |
|  |  | f__Nannocystaceae__uncultured |
|  |  | g__Variovorax |
|  |  | g__Caloramator |
|  |  | g__Thermoanaerobacterium |
|  |  | g__Methyloversatilis |
|  |  | g__mle1-27 |
|  |  | g__Microlunatus |
|  |  | g__Kribbella |
|  |  | g__Agromyces |
|  |  | g__Perlucidibaca |
|  |  | g__LiUU-11-161 |
|  |  | g__Candidatus_Solibacter |
|  |  | g__Jeotgalibaca |
|  |  | g__Blastomonas |
|  |  | g__Bacteroidales_RF16_group |
|  |  | g__Clostridia_vadinBB60_group |
|  |  | g__Pedomicrobium |
|  |  | g__Moryella |
|  |  | g__Rhodobacter |
|  |  | g__Hungateiclostridium |
|  |  | f__Sphingobacteriaceae |
|  |  | g__Burkholderia-Caballeronia-Paraburkholderia |
|  |  | g__Kibdelosporangium |
|  |  | g__IheB3-7 |
|  |  | p__Proteobacteria |
|  |  | g__1-20 |
|  |  | g__Thermicanus |
|  |  | g__Dechloromonas |
|  |  | g__WCHB1-41 |
|  |  | g__KF-JG30-B3 |
|  |  | g__Pseudorhodoferax |
|  |  | g__Fimbriimonadaceae |
|  |  | g__Nibribacter |
|  |  | g__Tissierella |
|  |  | g__Sumerlaea |
|  |  | g__Lechevalieria |
|  |  | g__D05-2 |
|  |  | g__Catellatospora |
|  |  | g__type_III |
|  |  | g__Rickettsiella |
|  |  | g__Arcanobacterium |
|  |  | g__env.OPS_17 |
|  |  | g__Planctomicrobium |
|  |  | g__Alloiococcus |
|  |  | o__Babeliales |
|  |  | g__Sphingoaurantiacus |
|  |  | f__Methylopilaceae |
|  |  | f__Pirellulaceae__uncultured |
|  |  | g__Blastocatella |
|  |  | g__Rhodoplanes |
|  |  | g__Thermosinus |
|  |  | f__Eggerthellaceae |
|  |  | g__Azospira |
|  |  | g__Novibacillus |
|  |  | g__Tuwongella |
|  |  | g__IMCC26256 |
|  |  | o__Erysipelotrichales__uncultured |
|  |  | g__Thermus |
|  |  | g__Acetitomaculum |
|  |  | g__Elusimicrobium |
|  |  | g__Kineosporia |
|  |  | g__Paludibacter |
|  |  | g__Phaselicystis |
|  |  | g__dgA-11_gut_group |
|  |  | g__Lachnospiraceae_UCG-010 |
|  |  | g__Sorangium |
|  |  | g__SZB85 |
|  |  | c__Clostridia__uncultured |
|  |  | g__CAP-aah99b04 |
|  |  | f__Xanthobacteraceae |
| Spring | 64 | g__Williamsia |
|  |  | g__Sphingorhabdus |
|  |  | g__Aestuariicella |
|  |  | g__Bacteroidales_UCG-001 |
|  |  | g__Confluentibacter |
|  |  | g__Algoriphagus |
|  |  | g__A4b |
|  |  | g__Blautia |
|  |  | g__Pontibacillus |
|  |  | g__Prevotellaceae_UCG-004 |
|  |  | g__Micromonospora |
|  |  | g__LWQ8 |
|  |  | Spring |
|  |  | g__Vitellibacter |
|  |  | g__Phascolarctobacterium |
|  |  | g__Brevibacillus |
|  |  | o__Microtrichales |
|  |  | g__Actinotalea |
|  |  | g__Kandleria |
|  |  | g__Halobacillus |
|  |  | g__Cellvibrio |
|  |  | g__Serratia |
|  |  | g__Kurthia |
|  |  | g__Gracilibacillus |
|  |  | f__Sandaracinaceae__uncultured |
|  |  | g__MWH-CFBk5 |
|  |  | g__R7C24 |
|  |  | f__Methylophilaceae |
|  |  | g__[Eubacterium]_xylanophilum_group |
|  |  | g__Clostridium_sensu_stricto_3 |
|  |  | f__Dermatophilaceae |
|  |  | g__Leucobacter |
|  |  | g__SWB02 |
|  |  | g__Tumebacillus |
|  |  | g__Carnobacterium |
|  |  | g__Myxococcus |
|  |  | g__Luteitalea |
|  |  | f__Chroococcidiopsaceae__uncultured |
|  |  | g__Marinicella |
|  |  | g__Ornithinicoccus |
|  |  | g__Ruminococcus |
|  |  | g__UCG-009 |
|  |  | g__Ammoniphilus |
|  |  | g__Pirellula |
|  |  | g__Tessaracoccus |
|  |  | g__Ornithobacterium |
|  |  | g__Myroides |
|  |  | g__Salinimicrobium |
|  |  | g__Brochothrix |
|  |  | g__Fibrella |
|  |  | g__HAW-RM37-2 |
|  |  | g__Demequina |
|  |  | g__Weissella |
|  |  | g__Psychrobacillus |
|  |  | g__Mobilicoccus |
|  |  | g__Succiniclasticum |
|  |  | f__Ardenticatenaceae__uncultured |
|  |  | g__Tomitella |
|  |  | f__Erwiniaceae |
|  |  | g__Crossiella |
|  |  | o__Micrococcales |
|  |  | g__Cereibacter |
|  |  | g__Reyranella |
|  |  | g__Raineyella |
| Summer | 83 | f__Comamonadaceae__uncultured |
|  |  | g__Paludisphaera |
|  |  | f__Myxococcaceae |
|  |  | c__Actinobacteria |
|  |  | g__Atopobium |
|  |  | g__Bauldia |
|  |  | f__Sphingomonadaceae__uncultured |
|  |  | f__Methanobacteriaceae |
|  |  | g__Lineage_IIb |
|  |  | g__[Anaerorhabdus]_furcosa_group |
|  |  | g__Prauserella |
|  |  | g__Fenollaria |
|  |  | g__Granulicatella |
|  |  | g__Actinocorallia |
|  |  | g__Abiotrophia |
|  |  | g__F0332 |
|  |  | g__Proteiniclasticum |
|  |  | g__Nakamurella |
|  |  | g__Pseudoalteromonas |
|  |  | g__p-251-o5 |
|  |  | g__Jatrophihabitans |
|  |  | g__Aridibacter |
|  |  | f__Eggerthellaceae__uncultured |
|  |  | f__Polyangiaceae |
|  |  | g__Marvinbryantia |
|  |  | g__Phocaeicola |
|  |  | g__Oribacterium |
|  |  | g__GCA-900066575 |
|  |  | g__Tepidisphaera |
|  |  | g__Akkermansia |
|  |  | g__JCM_18997 |
|  |  | g__Tychonema_CCAP_1459-11B |
|  |  | g__Domibacillus |
|  |  | g__Tepidisphaeraceae |
|  |  | g__Peptostreptococcus |
|  |  | f__Oscillospiraceae__uncultured |
|  |  | g__Dysgonomonas |
|  |  | g__Dermacoccus |
|  |  | g__Bergeriella |
|  |  | f__Xanthobacteraceae__uncultured |
|  |  | g__Motilibacter |
|  |  | f__Aeromonadaceae |
|  |  | g__Saccharothrix |
|  |  | g__Constrictibacter |
|  |  | g__Aerosphaera |
|  |  | g__Ohtaekwangia |
|  |  | g__CAG-352 |
|  |  | f__Verrucomicrobiaceae__uncultured |
|  |  | g__SH-PL14 |
|  |  | g__Capnocytophaga |
|  |  | g__IMCC26207 |
|  |  | p__Chloroflexi |
|  |  | g__Sulfurimonas |
|  |  | g__Comamonas |
|  |  | o__Thermomicrobiales |
|  |  | g__Anaerofustis |
|  |  | g__bacteriap25 |
|  |  | g__C0119 |
|  |  | g__Enterorhabdus |
|  |  | g__0319-7L14 |
|  |  | g__Taibaiella |
|  |  | f__Gemmatimonadaceae |
|  |  | f__Peptococcaceae__uncultured |
|  |  | f__Longimicrobiaceae |
|  |  | g__Alishewanella |
|  |  | f__Nitrososphaeraceae |
|  |  | c__Cyanobacteriia |
|  |  | g__Gitt-GS-136 |
|  |  | g__Campylobacter |
|  |  | g__Lachnospiraceae_XPB1014_group |
|  |  | g__TM7x |
|  |  | g__Family_XIII_UCG-001 |
|  |  | g__Tepidiphilus |
|  |  | g__11-24 |
|  |  | g__Azospirillum |
|  |  | g__Subdoligranulum |
|  |  | g__Dyadobacter |
|  |  | c__Alphaproteobacteria__uncultured |
|  |  | g__JGI_0000069-P22 |
|  |  | g__Antarcticibacterium |
|  |  | g__Lachnospiraceae_UCG-008 |
|  |  | g__Simonsiella |
|  |  | g__wb1-P19 |
