# Supplementary material for: Seasonal Variations in the Microbiome of Hyalomma excavatum Ticks in Algeria
Source: Microb Ecol. 2025 Sep 30;88(1):96. doi: 10.1007/s00248-025-02597-y (PMC12484380; doi:10.1007/s00248-025-02597-y)
Supplement: Supplementary file 2 — Supplementary Material 2 (DOCX 16.1 KB) [file 248_2025_2597_MOESM2_ESM.docx]

**Supplementary Table S2:**  Seasonal variations in bacterial taxa abundance based on CLR-transformed values

| Season hvjhvjvjjvjvjvjv | asvID | f__Enterobacteriaceae | f__Yersiniaceae | g__Staphylococcus | g__Candidatus_Midichloria | g__Francisella | g__Veillonella | g__Pseudomonas | g__Acinetobacter |
| --- | --- | --- | --- | --- | --- | --- | --- | --- | --- |
| Spring | a7 | 7.8999301516661 | 1.88281628402863 | 12.0049501487956 | 2.31863219595696 | 15.1359818596533 | 0 | 0 | 0 |
|  | a8 | 0.319149953862549 | 0.807688989361752 | 5.93761787931916 | 0.00370442528442361 | 0.200646038561871 | 0 | 0 | 0 |
|  | a9 | 0.729158133485313 | 0.348597737375474 | 0.42155321556174 | 0.884338871432369 | 0.710562786182859 | 0 | 0 | 0 |
|  | a11 | 16.162291739607 | 13.7725018880819 | 15.9757970927295 | 13.5434553030984 | 15.8744145785058 | 0 | 0 | 0 |
|  | a12 | 16.9887500125375 | 12.2249013369576 | 15.8582355120058 | 14.7635102254637 | 14.2404271237218 | 0 | 0 | 0 |
|  | a13 | 14.7904834162066 | 9.98532279005856 | 15.2617178913907 | 12.3888677097063 | 16.8842036364761 | 0 | 0 | 0 |
|  | a14 | 14.9144029538294 | 12.6150396274336 | 15.3985171731208 | 11.2356570860992 | 14.836012758675 | 0 | 0 | 0 |
| Summer | a23 | 5.62554530790531 | 0 | 0 | 13.094589079667 | 14.5879645783239 | 3.38256860730733 | 1.28550498213416 | 4.55645059580938 |
|  | a24 | 0.171672242649546 | 0 | 0 | 16.3738728238551 | 10.3750784037288 | 8.92671667346653 | 7.98330787107516 | 7.83874568692557 |
|  | a35 | 1.00476224391733 | 0 | 0 | 0.619404077675352 | 5.95100874646502 | 6.03983182917955 | 4.71080969228459 | 7.28709602433564 |
|  | a38 | 0.586679767745034 | 0 | 0 | 9.14740412812655 | 7.62498978907301 | 7.56138519066039 | 8.47997530810392 | 12.9243904111875 |
|  | a41 | 3.66619585760179 | 0 | 0 | 9.80678812842655 | 6.62023770310491 | 0.684974785138925 | 5.71773655990533 | 10.0077143714792 |
|  | a43 | 0.425353670004885 | 0 | 0 | 10.3429644609948 | 7.74213659248642 | 6.02660428711437 | 5.80386738415912 | 7.68676115597518 |
| Autumn | a47 | 6.15537738811895 | 0.305617650025196 | 17.4553107537359 | 8.8958398898377 | 0 | 0.0883415729210135 | 9.75063984738787 | 8.53941150734419 |
|  | a48 | 8.40914211487254 | 5.80421275407367 | 11.8556853503527 | 5.38354895238839 | 0 | 0.195164385642563 | 8.73521760182189 | 8.1424862572121 |
|  | a49 | 5.10943770384834 | 1.59603061850454 | 12.4012869849269 | 4.62365885010233 | 0 | 2.21725892910766 | 15.1677040668223 | 11.4963025762011 |
|  | a50 | 5.25902617444568 | 1.09879345971203 | 15.8536169980206 | 8.55430848510495 | 0 | 3.03101246624414 | 15.7101147987494 | 13.6046239623867 |
|  | a56 | 8.18369370130437 | 7.3587934846268 | 13.2500431751572 | 0.38133237196304 | 0 | 0.64776720514628 | 10.5829263101591 | 10.8684701802402 |
|  | a58 | 0.126850259458532 | 0.418093530939215 | 11.9163641663786 | 0.101846060630372 | 0 | 0.103656585456772 | 9.12704032323114 | 12.9620531955428 |
|  | a59 | 5.15874914989912 | 1.09441028192518 | 10.83441426648 | 9.69686622004104 | 0 | 0.851952446200466 | 11.6505407306055 | 14.3088725779018 |
|  | a60 | 5.29508521636363 | 2.94928227323855 | 8.63058891173367 | 3.89271660911248 | 0 | 2.6077259395693 | 11.7765365816512 | 11.7125421983456 |
