# Supplementary material for: Seasonal Variations in the Microbiome of Hyalomma excavatum Ticks in Algeria
Source: Microb Ecol. 2025 Sep 30;88(1):96. doi: 10.1007/s00248-025-02597-y (PMC12484380; doi:10.1007/s00248-025-02597-y)
Supplement: Supplementary file 3 — Supplementary Material 3 (DOCX 27.2 KB) [file 248_2025_2597_MOESM3_ESM.docx]

**Supplementary Table S3:** Shared and unique microbial taxa across seasonal networks (GN, NWoF, NWoR)

| Names | Total | Elements |
| --- | --- | --- |
| Autumn AutumnWithoutFrancisella AutumnWithoutRickettsia | 126 | g__Streptomyces |
|  |  | g__Massilia |
|  |  | g__Segetibacter |
|  |  | g__Burkholderia-Caballeronia-Paraburkholderia |
|  |  | f__Comamonadaceae |
|  |  | f__Lachnospiraceae |
|  |  | g__IheB3-7 |
|  |  | g__Vulcaniibacterium |
|  |  | g__Arthrobacter |
|  |  | g__Nocardioides |
|  |  | g__Pseudonocardia |
|  |  | g__Lachnospiraceae_NK3A20_group |
|  |  | g__Pseudomonas |
|  |  | g__Geodermatophilus |
|  |  | g__Bergeyella |
|  |  | f__Pseudomonadaceae |
|  |  | f__Planococcaceae |
|  |  | g__Paracoccus |
|  |  | g__Thermicanus |
|  |  | g__Hydrogenophilus |
|  |  | g__Moraxella |
|  |  | g__Mycobacterium |
|  |  | g__Aeromonas |
|  |  | f__Caloramatoraceae |
|  |  | g__Cellulomonas |
|  |  | g__Candidatus_Saccharimonas |
|  |  | g__Micrococcus |
|  |  | f__Beijerinckiaceae |
|  |  | g__Treponema |
|  |  | g__Pseudorhodoferax |
|  |  | g__Hymenobacter |
|  |  | g__Sphingobium |
|  |  | f__Beijerinckiaceae__uncultured |
|  |  | o__Enterobacterales |
|  |  | f__Selenomonadaceae |
|  |  | g__Thermonema |
|  |  | f__Kineosporiaceae |
|  |  | g__67-14 |
|  |  | g__Paenarthrobacter |
|  |  | g__Legionella |
|  |  | o__Bacillales |
|  |  | f__Ruminococcaceae |
|  |  | g__Pantoea |
|  |  | f__Xanthomonadaceae |
|  |  | o__Planctomycetales__uncultured |
|  |  | g__Noviherbaspirillum |
|  |  | g__Lysinibacillus |
|  |  | g__Tepidimonas |
|  |  | g__Aquabacterium |
|  |  | g__Methylobacterium-Methylorubrum |
|  |  | g__Roseisolibacter |
|  |  | f__Isosphaeraceae |
|  |  | g__Rubrobacter |
|  |  | g__Microvirga |
|  |  | g__Adhaeribacter |
|  |  | g__Xanthomonas |
|  |  | g__Phyllobacterium |
|  |  | g__Negativicoccus |
|  |  | g__Roseomonas |
|  |  | g__Rheinheimera |
|  |  | g__Flavobacterium |
|  |  | g__Arcanobacterium |
|  |  | f__Yersiniaceae |
|  |  | g__Solirubrobacter |
|  |  | g__Chryseobacterium |
|  |  | g__Muribaculum |
|  |  | f__Aerococcaceae__uncultured |
|  |  | c__Clostridia |
|  |  | g__Actinomycetospora |
|  |  | g__Bacteroides |
|  |  | g__Clostridioides |
|  |  | g__Christensenellaceae_R-7_group |
|  |  | g__Cutibacterium |
|  |  | g__Cardiobacterium |
|  |  | g__Microbacterium |
|  |  | g__Romboutsia |
|  |  | g__Fonticella |
|  |  | f__Ilumatobacteraceae__uncultured |
|  |  | f__Rhodocyclaceae |
|  |  | g__Luteimonas |
|  |  | g__Fervidobacterium |
|  |  | g__Thermosinus |
|  |  | f__Neisseriaceae__uncultured |
|  |  | f__Eggerthellaceae |
|  |  | g__Brevundimonas |
|  |  | g__Blastococcus |
|  |  | o__Frankiales__uncultured |
|  |  | g__Nocardiopsis |
|  |  | g__Rubellimicrobium |
|  |  | g__Candidatus_Alysiosphaera |
|  |  | g__Craurococcus-Caldovatus |
|  |  | g__Sphingomonas |
|  |  | g__Bosea |
|  |  | g__Actinomyces |
|  |  | f__Isosphaeraceae__uncultured |
|  |  | g__Fusobacterium |
|  |  | g__Sporosarcina |
|  |  | g__Caloramator |
|  |  | g__Haemophilus |
|  |  | g__Thermoanaerobacterium |
|  |  | g__Caldibacillus |
|  |  | g__Methyloversatilis |
|  |  | g__Thermus |
|  |  | g__Enhydrobacter |
|  |  | g__Altererythrobacter |
|  |  | g__Geobacillus |
|  |  | f__Gemmataceae__uncultured |
|  |  | g__Vogesella |
|  |  | g__Allorhizobium-Neorhizobium-Pararhizobium-Rhizobium |
|  |  | g__Prevotella |
|  |  | g__Corynebacterium |
|  |  | g__Anoxybacillus |
|  |  | g__[Eubacterium]_coprostanoligenes_group |
|  |  | g__Lawsonella |
|  |  | g__Helcococcus |
|  |  | g__Family_XIII_AD3011_group |
|  |  | g__Rhodococcus |
|  |  | g__Modestobacter |
|  |  | g__Bradyrhizobium |
|  |  | g__JG30-KF-CM45 |
|  |  | d__Bacteria |
|  |  | g__Staphylococcus |
|  |  | f__Microbacteriaceae |
|  |  | f__Micrococcaceae |
|  |  | g__Rhodobacter |
|  |  | g__Kocuria |
| Autumn AutumnWithoutFrancisella | 7 | g__0319-6G20 |
|  |  | g__Mogibacterium |
|  |  | g__Sumerlaea |
|  |  | g__Paenibacillus |
|  |  | g__Rickettsia |
|  |  | g__Trueperella |
|  |  | g__Gemella |
| Autumn AutumnWithoutRickettsia | 5 | g__Francisella |
|  |  | g__RB41 |
|  |  | g__Erysipelotrichaceae_UCG-009 |
|  |  | f__Erysipelotrichaceae__uncultured |
|  |  | g__Deinococcus |
| AutumnWithoutFrancisella AutumnWithoutRickettsia | 9 | g__Marmoricola |
|  |  | g__Riemerella |
|  |  | g__Saccharimonadales |
|  |  | f__Oscillospiraceae |
|  |  | g__Marisediminicola |
|  |  | p__Armatimonadota__uncultured |
|  |  | g__Microlunatus |
|  |  | f__Solirubrobacteraceae |
|  |  | g__WD2101_soil_group |
| Autumn | 6 | g__JG30-KF-CM66 |
|  |  | g__Candidatus_Peribacteria |
|  |  | g__Opitutus |
|  |  | g__Pir4_lineage |
|  |  | g__Flexilinea |
|  |  | g__Moryella |
| AutumnWithoutFrancisella | 9 | g__Subgroup_7 |
|  |  | g__Leptotrichia |
|  |  | g__Kineococcus |
|  |  | o__Solirubrobacterales |
|  |  | g__Porphyromonas |
|  |  | g__Agromyces |
|  |  | g__Friedmanniella |
|  |  | g__Longimicrobium |
|  |  | f__Hyphomicrobiaceae |
| AutumnWithoutRickettsia | 16 | g__Saccharimonadaceae |
|  |  | g__Sphingopyxis |
|  |  | g__Candidatus_Protochlamydia |
|  |  | g__Exiguobacterium |
|  |  | o__Vicinamibacterales__uncultured |
|  |  | g__Bibersteinia |
|  |  | g__Methanosphaera |
|  |  | c__Acidimicrobiia__uncultured |
|  |  | g__Promicromonospora |
|  |  | g__Cryptosporangium |
|  |  | g__Chthoniobacter |
|  |  | g__Acetitomaculum |
|  |  | g__Acinetobacter |
|  |  | g__Olsenella |
|  |  | g__Rothia |
|  |  | f__Sporichthyaceae__uncultured |

| Names | total | elements |
| --- | --- | --- |
| Spring SpringWithoutFrancisella | 10 | g__Pseudomonas |
|  |  | f__Planococcaceae |
|  |  | g__Aeromonas |
|  |  | g__Pantoea |
|  |  | g__Lysinibacillus |
|  |  | g__Paenibacillus |
|  |  | g__Sporosarcina |
|  |  | g__Staphylococcus |
|  |  | d__Bacteria |
|  |  | g__Kocuria |
| Spring | 134 | g__Streptomyces |
|  |  | g__Massilia |
|  |  | g__Segetibacter |
|  |  | g__Burkholderia-Caballeronia-Paraburkholderia |
|  |  | f__Comamonadaceae |
|  |  | g__0319-6G20 |
|  |  | f__Lachnospiraceae |
|  |  | g__IheB3-7 |
|  |  | g__Vulcaniibacterium |
|  |  | g__Arthrobacter |
|  |  | g__Nocardioides |
|  |  | g__JG30-KF-CM66 |
|  |  | g__Pseudonocardia |
|  |  | g__Lachnospiraceae_NK3A20_group |
|  |  | g__Bergeyella |
|  |  | g__Geodermatophilus |
|  |  | f__Pseudomonadaceae |
|  |  | g__Candidatus_Peribacteria |
|  |  | g__Paracoccus |
|  |  | g__Thermicanus |
|  |  | g__Hydrogenophilus |
|  |  | g__Moraxella |
|  |  | g__Mycobacterium |
|  |  | f__Caloramatoraceae |
|  |  | g__Cellulomonas |
|  |  | g__Candidatus_Saccharimonas |
|  |  | g__Micrococcus |
|  |  | f__Beijerinckiaceae |
|  |  | g__Pseudorhodoferax |
|  |  | g__Treponema |
|  |  | g__Hymenobacter |
|  |  | g__Sphingobium |
|  |  | g__Francisella |
|  |  | f__Beijerinckiaceae__uncultured |
|  |  | o__Enterobacterales |
|  |  | f__Selenomonadaceae |
|  |  | g__Thermonema |
|  |  | f__Kineosporiaceae |
|  |  | g__67-14 |
|  |  | g__Paenarthrobacter |
|  |  | g__Legionella |
|  |  | o__Bacillales |
|  |  | f__Ruminococcaceae |
|  |  | g__Mogibacterium |
|  |  | f__Xanthomonadaceae |
|  |  | o__Planctomycetales__uncultured |
|  |  | g__Noviherbaspirillum |
|  |  | g__Sumerlaea |
|  |  | g__Tepidimonas |
|  |  | g__Aquabacterium |
|  |  | g__Methylobacterium-Methylorubrum |
|  |  | f__Isosphaeraceae |
|  |  | g__Roseisolibacter |
|  |  | g__Rubrobacter |
|  |  | g__Adhaeribacter |
|  |  | g__Microvirga |
|  |  | g__Xanthomonas |
|  |  | g__RB41 |
|  |  | g__Phyllobacterium |
|  |  | g__Negativicoccus |
|  |  | g__Roseomonas |
|  |  | g__Rheinheimera |
|  |  | g__Flavobacterium |
|  |  | g__Arcanobacterium |
|  |  | f__Yersiniaceae |
|  |  | g__Opitutus |
|  |  | g__Solirubrobacter |
|  |  | g__Chryseobacterium |
|  |  | g__Muribaculum |
|  |  | f__Aerococcaceae__uncultured |
|  |  | c__Clostridia |
|  |  | g__Actinomycetospora |
|  |  | g__Bacteroides |
|  |  | g__Clostridioides |
|  |  | g__Christensenellaceae_R-7_group |
|  |  | g__Cutibacterium |
|  |  | g__Microbacterium |
|  |  | g__Erysipelotrichaceae_UCG-009 |
|  |  | g__Cardiobacterium |
|  |  | g__Romboutsia |
|  |  | g__Fonticella |
|  |  | f__Ilumatobacteraceae__uncultured |
|  |  | f__Rhodocyclaceae |
|  |  | g__Luteimonas |
|  |  | g__Fervidobacterium |
|  |  | f__Erysipelotrichaceae__uncultured |
|  |  | g__Thermosinus |
|  |  | f__Neisseriaceae__uncultured |
|  |  | f__Eggerthellaceae |
|  |  | g__Brevundimonas |
|  |  | g__Blastococcus |
|  |  | o__Frankiales__uncultured |
|  |  | g__Nocardiopsis |
|  |  | g__Rubellimicrobium |
|  |  | g__Candidatus_Alysiosphaera |
|  |  | g__Deinococcus |
|  |  | g__Sphingomonas |
|  |  | g__Craurococcus-Caldovatus |
|  |  | g__Bosea |
|  |  | f__Isosphaeraceae__uncultured |
|  |  | g__Actinomyces |
|  |  | g__Fusobacterium |
|  |  | g__Rickettsia |
|  |  | g__Caloramator |
|  |  | g__Haemophilus |
|  |  | g__Thermoanaerobacterium |
|  |  | g__Caldibacillus |
|  |  | g__Methyloversatilis |
|  |  | g__Thermus |
|  |  | g__Enhydrobacter |
|  |  | g__Altererythrobacter |
|  |  | g__Geobacillus |
|  |  | f__Gemmataceae__uncultured |
|  |  | g__Vogesella |
|  |  | g__Prevotella |
|  |  | g__Allorhizobium-Neorhizobium-Pararhizobium-Rhizobium |
|  |  | g__Corynebacterium |
|  |  | g__Pir4_lineage |
|  |  | g__Flexilinea |
|  |  | g__Anoxybacillus |
|  |  | g__Lawsonella |
|  |  | g__[Eubacterium]_coprostanoligenes_group |
|  |  | g__Helcococcus |
|  |  | g__Rhodococcus |
|  |  | g__Family_XIII_AD3011_group |
|  |  | g__Modestobacter |
|  |  | g__Bradyrhizobium |
|  |  | g__Trueperella |
|  |  | g__JG30-KF-CM45 |
|  |  | f__Microbacteriaceae |
|  |  | f__Micrococcaceae |
|  |  | g__Moryella |
|  |  | g__Gemella |
|  |  | g__Rhodobacter |
| SpringWithoutFrancisella | 21 | g__Bacillus |
|  |  | g__Candidatus_Midichloria |
|  |  | g__Serratia |
|  |  | g__Kurthia |
|  |  | g__Psychrobacillus |
|  |  | f__Pasteurellaceae |
|  |  | g__Virgibacillus |
|  |  | g__Bibersteinia |
|  |  | f__Enterobacteriaceae |
|  |  | g__Leuconostoc |
|  |  | g__Shewanella |
|  |  | g__Psychrobacter |
|  |  | g__Mitochondria |
|  |  | g__Lactococcus |
|  |  | g__Myroides |
|  |  | g__Brochothrix |
|  |  | f__Erwiniaceae |
|  |  | g__Chloroplast |
|  |  | g__Acinetobacter |
|  |  | g__Weissella |
|  |  | o__Lactobacillales |

| Names | total | elements |
| --- | --- | --- |
| Summer SummerWithoutFrancisella SummerWithoutRickettsia | 129 | g__Massilia |
|  |  | f__Carnobacteriaceae |
|  |  | f__Carnobacteriaceae__uncultured |
|  |  | g__Bacillus |
|  |  | g__Segetibacter |
|  |  | f__Neisseriaceae |
|  |  | g__Mannheimia |
|  |  | g__Patulibacter |
|  |  | g__Cnuella |
|  |  | f__Comamonadaceae |
|  |  | g__Aridibacter |
|  |  | g__Vulcaniibacterium |
|  |  | g__Cloacibacterium |
|  |  | g__Arthrobacter |
|  |  | g__Stenotrophomonas |
|  |  | g__Nocardioides |
|  |  | g__Pedobacter |
|  |  | g__Pseudonocardia |
|  |  | g__Bergeyella |
|  |  | g__Geodermatophilus |
|  |  | g__Bifidobacterium |
|  |  | f__Planococcaceae |
|  |  | g__Veillonella |
|  |  | g__Exiguobacterium |
|  |  | g__Hydrogenophilus |
|  |  | g__Moraxella |
|  |  | g__Mycobacterium |
|  |  | g__Thermomonas |
|  |  | g__Schlegelella |
|  |  | g__Neisseria |
|  |  | g__Aeromonas |
|  |  | f__Aeromonadaceae |
|  |  | f__Geodermatophilaceae |
|  |  | g__Cellulomonas |
|  |  | g__Candidatus_Saccharimonas |
|  |  | f__Pasteurellaceae |
|  |  | g__Micrococcus |
|  |  | g__Bauldia |
|  |  | g__Hymenobacter |
|  |  | f__Methanobacteriaceae |
|  |  | f__Kineosporiaceae |
|  |  | g__67-14 |
|  |  | g__Methanobrevibacter |
|  |  | f__Ruminococcaceae |
|  |  | g__Mogibacterium |
|  |  | g__Virgibacillus |
|  |  | g__Brevibacterium |
|  |  | g__Marmoricola |
|  |  | g__Bibersteinia |
|  |  | g__Lysinibacillus |
|  |  | g__Coprococcus |
|  |  | g__Riemerella |
|  |  | f__Ruminococcaceae__uncultured |
|  |  | f__Eggerthellaceae__uncultured |
|  |  | g__Ferruginibacter |
|  |  | g__Rubrobacter |
|  |  | g__Adhaeribacter |
|  |  | g__Xanthomonas |
|  |  | g__Atopostipes |
|  |  | g__Rheinheimera |
|  |  | f__Lachnospiraceae__uncultured |
|  |  | g__Streptococcus |
|  |  | f__Aerococcaceae |
|  |  | g__Leuconostoc |
|  |  | f__Longimicrobiaceae |
|  |  | g__Phocaeicola |
|  |  | g__Escherichia-Shigella |
|  |  | g__Anaerococcus |
|  |  | g__Dietzia |
|  |  | g__Christensenellaceae_R-7_group |
|  |  | g__Cutibacterium |
|  |  | g__NK4A214_group |
|  |  | g__Oribacterium |
|  |  | g__Romboutsia |
|  |  | g__Campylobacter |
|  |  | g__Citricoccus |
|  |  | g__CAG-352 |
|  |  | f__Acetobacteraceae |
|  |  | g__Belnapia |
|  |  | g__GCA-900066575 |
|  |  | f__Neisseriaceae__uncultured |
|  |  | g__Brachybacterium |
|  |  | g__Fenollaria |
|  |  | g__Blastococcus |
|  |  | g__Actinocorallia |
|  |  | g__Alloprevotella |
|  |  | g__Quadrisphaera |
|  |  | g__Nocardiopsis |
|  |  | g__Rubellimicrobium |
|  |  | g__PMMR1 |
|  |  | g__Abiotrophia |
|  |  | g__Sphingomonas |
|  |  | g__Actinomyces |
|  |  | g__Fusobacterium |
|  |  | g__Porphyromonas |
|  |  | g__Alysiella |
|  |  | g__Lautropia |
|  |  | g__F0332 |
|  |  | g__Enhydrobacter |
|  |  | g__Lactobacillus |
|  |  | g__Subdoligranulum |
|  |  | g__Geobacillus |
|  |  | g__Sphingobacterium |
|  |  | g__Peptostreptococcus |
|  |  | g__Friedmanniella |
|  |  | g__Vogesella |
|  |  | g__Prevotella |
|  |  | g__Allorhizobium-Neorhizobium-Pararhizobium-Rhizobium |
|  |  | f__Oscillospiraceae__uncultured |
|  |  | g__Acinetobacter |
|  |  | g__Gracilibacteria |
|  |  | g__Aerococcus |
|  |  | g__Lawsonella |
|  |  | g__Helcococcus |
|  |  | g__Rothia |
|  |  | g__Antarcticibacterium |
|  |  | g__Modestobacter |
|  |  | g__Dermacoccus |
|  |  | g__Trueperella |
|  |  | g__JG30-KF-CM45 |
|  |  | g__AKIW781 |
|  |  | g__Mesorhizobium |
|  |  | g__Simonsiella |
|  |  | g__Enterococcus |
|  |  | g__Pseudoalteromonas |
|  |  | g__p-251-o5 |
|  |  | f__Micrococcaceae |
|  |  | g__Jatrophihabitans |
|  |  | g__Gemella |
| Summer SummerWithoutFrancisella | 20 | o__Bacillales |
|  |  | f__Blastocatellaceae__uncultured |
|  |  | g__Methylobacterium-Methylorubrum |
|  |  | g__Roseisolibacter |
|  |  | o__Microtrichales__uncultured |
|  |  | c__Clostridia |
|  |  | g__Prauserella |
|  |  | o__Frankiales__uncultured |
|  |  | g__Lachnospiraceae_XPB1014_group |
|  |  | g__Rickettsia |
|  |  | g__Family_XIII_UCG-001 |
|  |  | g__RF39 |
|  |  | o__Frankiales |
|  |  | g__WD2101_soil_group |
|  |  | c__Alphaproteobacteria__uncultured |
|  |  | g__Vicinamibacteraceae |
|  |  | g__Longimicrobium |
|  |  | g__Family_XIII_AD3011_group |
|  |  | g__Clostridia_UCG-014 |
|  |  | g__Aureimonas |
| Summer SummerWithoutRickettsia | 10 | g__Taibaiella |
|  |  | g__Francisella |
|  |  | f__Isosphaeraceae |
|  |  | g__RB41 |
|  |  | g__Devosia |
|  |  | g__Paenibacillus |
|  |  | g__Craurococcus-Caldovatus |
|  |  | g__Truepera |
|  |  | g__Clostridium_sensu_stricto_1 |
|  |  | f__Rhodobacteraceae |
| SummerWithoutFrancisella SummerWithoutRickettsia | 11 | g__Amaricoccus |
|  |  | f__Thermomonosporaceae |
|  |  | g__Turicibacter |
|  |  | g__Noviherbaspirillum |
|  |  | g__Leptotrichia |
|  |  | g__Roseomonas |
|  |  | f__Blastocatellaceae |
|  |  | g__[Anaerorhabdus]_furcosa_group |
|  |  | g__Hydrogenophaga |
|  |  | g__Tepidiphilus |
|  |  | g__Rhodococcus |
| Summer | 10 | g__Lachnospiraceae_NK3A20_group |
|  |  | f__Aerococcaceae__uncultured |
|  |  | g__Skermanella |
|  |  | g__Actinomycetospora |
|  |  | f__Verrucomicrobiaceae__uncultured |
|  |  | f__Erysipelotrichaceae__uncultured |
|  |  | g__Candidatus_Alysiosphaera |
|  |  | f__Bacillaceae |
|  |  | g__Dyadobacter |
|  |  | f__Sporichthyaceae__uncultured |
| SummerWithoutFrancisella | 13 | g__0319-7L14 |
|  |  | f__Beijerinckiaceae__uncultured |
|  |  | f__Enterobacteriaceae |
|  |  | g__Microvirga |
|  |  | g__Marvinbryantia |
|  |  | f__Gemmatimonadaceae |
|  |  | g__Flavobacterium |
|  |  | f__Micromonosporaceae |
|  |  | g__Akkermansia |
|  |  | g__TM7x |
|  |  | g__Domibacillus |
|  |  | g__Anaerofustis |
|  |  | f__Microbacteriaceae |
| SummerWithoutRickettsia | 22 |  |
|  |  | f__Frankiales |
|  |  | f__WD2101_soil_group |
|  |  | f__Lachnospiraceae |
|  |  | g__Alphaproteobacteria__uncultured |
|  |  | f__Myxococcaceae |
|  |  | f__Microtrichales__uncultured |
|  |  | g__Candidatus_Nitrocosmicus |
|  |  | f__Bacillales |
|  |  | f__Saccharimonadales |
|  |  | f__Lachnospiraceae_XPB1014_group |
|  |  | f__Frankiales__uncultured |
|  |  | f__RF39 |
|  |  | f__Clostridia_UCG-014 |
|  |  | f__Family_XIII_UCG-001 |
|  |  | g__Tychonema_CCAP_1459-11B |
|  |  | f__Lachnospiraceae_NK3A20_group |
|  |  | g__Conexibacter |
|  |  | g__Anoxybacillus |
|  |  | f__Family_XIII_AD3011_group |
|  |  | f__Clostridia |
|  |  | f__Vicinamibacteraceae |
